# Ornithopod diversity in the Griman Creek Formation (Cenomanian), New South Wales, Australia

**DOI:** 10.7717/peerj.6008

**Published:** 2018-12-04

**Authors:** Phil R. Bell, Matthew C. Herne, Tom Brougham, Elizabeth T. Smith

**Affiliations:** 1 School of Environmental and Rural Science, University of New England, Armidale, NSW, Australia; 2 School of Biological Sciences, University of Queensland, Brisbane, QLD, Australia; 3 Australian Opal Centre, Lightning Ridge, NSW, Australia

**Keywords:** Cretaceous, Dinosauria, Ornithischia, Ornithopoda, Griman creek formation, Australia

## Abstract

During the Early Cretaceous, dinosaur communities of the Australian-Antarctic rift system (Eumeralla and Wonthaggi formations) cropping out in Victoria were apparently dominated by a diverse small-bodied ‘basal ornithopod’ fauna. Further north, in Queensland (Winton and Mackunda formations), poorly-represented small-bodied ornithopods coexisted with large-bodied iguanodontians. Our understanding of the ornithopod diversity from the region between the Australian-Antarctic rift and Queensland, represented by Lightning Ridge in central-northern New South Wales (Griman Creek Formation), has been superficial. Here, we re-investigate the ornithopod diversity at Lightning Ridge based on new craniodental remains. Our findings indicate a diverse ornithopod fauna consisting of two-to-three small-bodied non-iguanodontian ornithopods (including *Weewarrasaurus pobeni* gen. et sp. nov.), at least one indeterminate iguanodontian, and a possible ankylopollexian. These results support those of previous studies that favour a general abundance of small-bodied basal ornithopods in Early to mid-Cretaceous high-latitude localities of southeastern Australia. Although these localities are not necessarily time-equivalent, increasing evidence indicates that Lightning Ridge formed a ‘meeting point’ between the basal ornithopod-dominated localities in Victoria and the sauropod-iguanodontian faunas in Queensland to the north.

## Introduction

**Figure 1:**
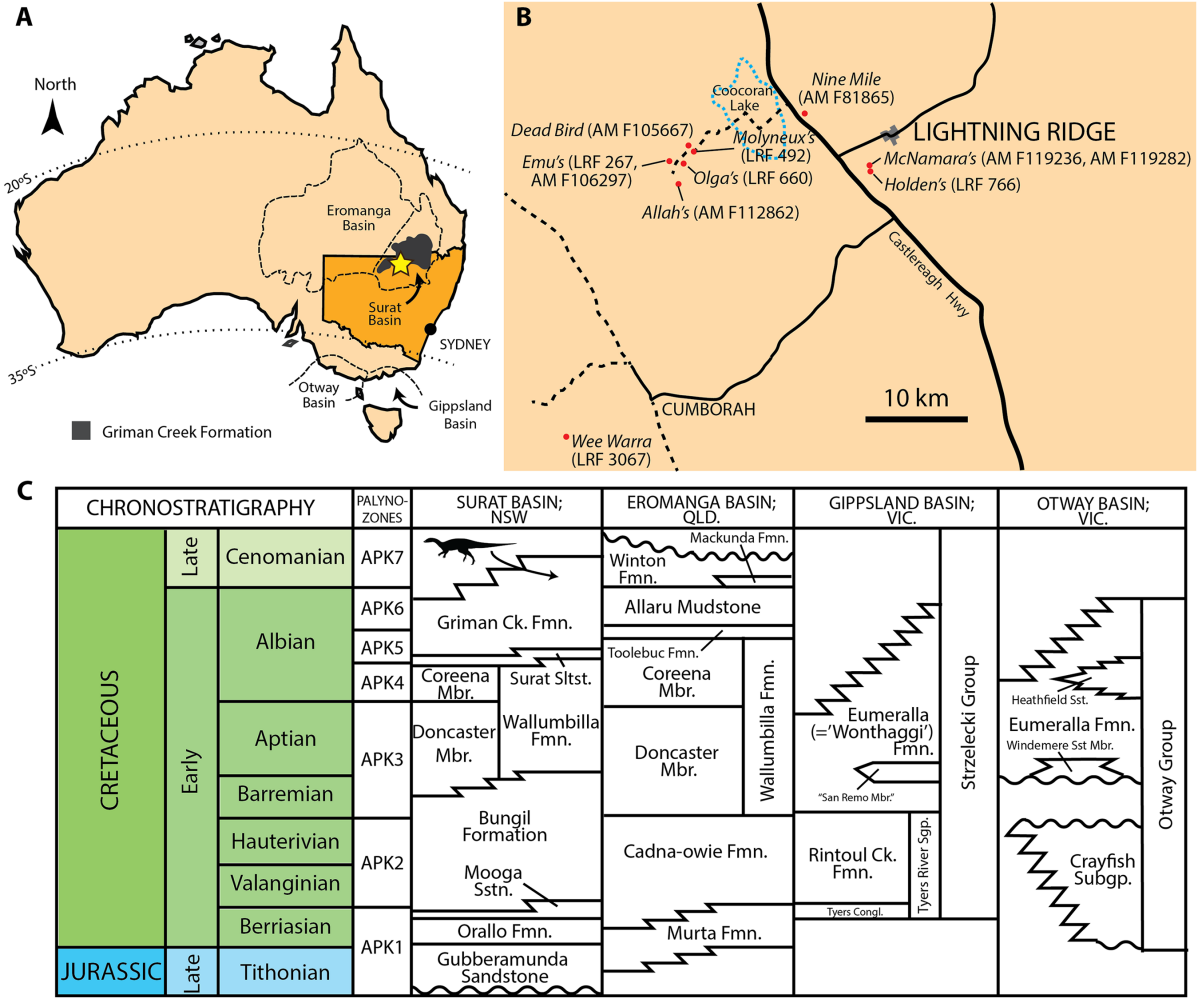
Mid-Cretaceous geology of Australia. (A) Present day map of Australia with the town of Lightning Ridge indicated by the star. (B) Regional map of the Lightning Ridge region showing localities (where known) for specimens described in this text. Sealed (solid black lines) and unsealed roads (dashed lines) are indicated. The ephemeral Coocoran Lake is marked with a dotted blue line. (C) Correlative stratigraphy of the major Cretaceous depositional basins and geological units discussed in this study. The ornithopod icon and arrow indicate the approximate level of the Griman Creek Formation from which the current material pertains. Informal units are in quotation marks. Maps in (A) and (B) redrawn and modified from Bell et al. (2016) and Opal Fields—Lightning Ridge Region map produced by the NSW Department of Mineral Resources, respectively. Stratigraphy based on Toslini, McLoughlin & Drinnan (1999) and Cook, Bryan & Draper (2013). Ornithopod silhouette created by Caleb M. Brown and used under the Creative Commons Attribution-ShareAlike 3.0 Unported license.

Small, bipedal neornithischian dinosaurs, traditionally referred to as ‘hypsilophodontids’ or ‘basal ornithopods’, are typically rare in most Cretaceous deposits, globally (Brown, Boyd & Russell, 2011; Brown et al., 2013). In contrast, the Aptian–Albian aged deposits of southeastern Australia have yielded an unusual preponderance of these enigmatic animals (Rich & Vickers-Rich, 1989, 1999). From the state of Victoria alone (Fig. 1C), four locally endemic genera have been described, including *Atlascopcosaurus loadsi* (Rich & Vickers-Rich, 1989)*, Diluvicursor pickeringi* (Herne et al., 2018), and *Leaellynasaura amicagraphica* (Rich & Vickers-Rich, 1989) from the lower Albian of the Eumeralla Formation (Otway Basin) and *Qantassaurus intrepidus* (Rich & Vickers-Rich, 1999) from the upper Aptian of the Wonthaggi Formation (Strzelecki Group of the Gippsland Basin). A fifth species (*Fulgurotherium australe*) that was originally described from the Griman Creek Formation (GCF) in central-northern New South Wales (Von Huene, 1932) was also identified in the Early Cretaceous deposits of Victoria (Rich & Vickers-Rich, 1989, 1999). However, *F. australe* is now considered a nomen dubium (Agnolin et al., 2010) and there is disagreement as to the validity of some of the other Victorian taxa, or materials assigned to some taxa (Herne & Salisbury, 2009; Agnolin et al., 2010; Rich, Galton & Vickers-Rich, 2010). Despite such disagreements, the Early Cretaceous of Victoria preserves a demonstrably diverse small-bodied ornithopod fauna (Herne, 2014), which contrasts with the sauropod-dominated Winton Formation in Queensland, where only a single ornithopod tooth has been described (Hocknull & Cook, 2008). Abundant ornithopod tracks, however, are also known from the Winton Formation (Thulborn & Wade, 1984; Romilio, Tucker & Salisbury, 2013). Similarly, the presence of both large and small-bodied ornithopods in the Valanginian–Barremian Broome Sandstone in Western Australia is established based on an extensive trackway record (Salisbury et al., 2017).

The only large-bodied ornithopod named from Australia is *Muttaburrasaurus langdoni*, which is usually considered a basal Iguanodontia (Norman, 2004; Boyd, 2015). The holotype of that taxon comes from the Mackunda Formation (Bartholomai & Molnar, 1981), which underlies the Winton Formation, although additional referred specimens (*Muttaburrasaurus* sp.; QM F14921 (the ‘Dunluce’ skull) and QM F12541) come from the slightly older Allaru Mudstone (Molnar, 1996). A second large-bodied iguanodontian has been identified from the GCF in northern New South Wales and is currently being described by the authors.

The GCF, part of the Surat Basin, is notable as the only dinosaur-bearing formation in New South Wales, cropping out near the town of Lightning Ridge in the central-northern part of the state (Fig. 1). Recent radiometric dating has revealed a maximum age of ∼96–100 Ma in the area, making the exposures at Lightning Ridge Cenomanian in age and approximately 10 Ma younger than equivalent exposures of the GCF in the northern (Queensland) part of the Surat Basin (Bell et al., 2016; Bell et al., 2019). At Lightning Ridge, small ornithopod bones dominate the dinosaur remains although dental and cranial elements are rare (P. Bell, 2018, E. Smith, 2018, personal observation). Ornithopod remains have been known from this formation since the description of ‘*Fulgurotherium*’ in 1932, which was based on an incomplete distal femur (although it was mistakenly identified as a theropod at the time (Von Huene, 1932)). The original interpretation was later corrected by Molnar (1980a), who also briefly described a number of other ornithopod elements from Lightning Ridge, including a tooth (QM F9505). Molnar & Galton (1986) evaluated seven small ornithopod femora from the GCF, recognising two distinct morphotypes. All of this material was assigned to ‘Hypsilophodontidae’ (Molnar, 1980a, Molnar & Galton, 1986). A *Muttaburrasaurus*-like species of iguanodontian was later recognised based on two isolated teeth (Molnar, 1996)—although only one specimen (QM F14421) was described—bringing the number of ornithopods from this interval to three.

The proliferation of new taxa (particularly within Asia and North and South America) combined with improved understandings of ornithischian systematics in recent years makes it possible to re-evaluate the diversity and relationships of the Griman Creek ornithopods. Here we describe new material and redescribe previously published ornithopod craniodental remains from the GCF with the aim of revealing the taxonomic diversity of this group.

## Materials and Methods

Specimens were scanned using a GE-Phoenix V|tome|xs μCT scanner at the University of New England (Armidale, NSW, Australia) and captured using Datos acquisition and reconstruction software v.2.2.1. RTM. Samples were mounted on a rotating stage and imaged using the predetermined optimal X-ray tube settings (160 kv; voxel size = 16.505619 μm; 333 ms integration time per projection). The tomographs were visualised in Mimics 19.0 at the Function Evolution Anatomy Research lab at the University of New England, where they were manually ‘dissected’ using the thresholding and mask tools. The raw μCT files are freely available from Figshare using the following DOIs: 10.6084/m9.figshare.6959372, 10.6084/m9.figshare.6959486, 10.6084/m9.figshare.6962816.

Several teeth were moulded using Pinkysil® fast set silicone and cast in Procast® medium set polyurethane. Casts were coated in ammonium chloride to increase contrast and photographed using a Canon EOS5D DSLR. Selected standard measurements were made using digital callipers and presented in Table 1.

**Table 1:** Selected measurements of ornithopod cranial and dental remains from the Griman Creek Formation.

Specimen No.	Element	Taxonomy	Locality	Measurements (mm)			
				CH	CL	Max L	Max W
LRF 0267	Basioccipital	Ankylopollexia indet.	?Emu’s	–	–	16.5	18.0
AM F119236; QM F14420 (cast)	Dentary crown and root	Iguanodontia indet	McNamara’s	–	–	–	–
AM F119282; QM F14421 (cast)	Maxillary crown and root	Iguanodontia indet	McNamara’s	10.4	14.4	–	–
AM F81865	Maxillary crown	Iguanodontia indet	Nine mile	19.9	14.9	–	–
LRF 492	Dentary crown and root	Iguanodontia indet	Molyneux	17.1	17.9	–	–
LRF R1556	Jugal	Iguanodontia indet	?	–	–	26.1	–
AM F112862	Dentary crown	Ornithopoda indet.	Allah’s	9.7	7.1	–	–
LRF 660	Premaxillary crown	Ornithopoda indet.	Olga’s	∼10	10.0	–	–
AM F105667	Partial dentary with dentition	Ornithopoda indet. A	Dead bird	–	–	17.3	–
AM F106297	Partial dentary with dentition	Ornithopoda indet. A	Emu’s	–	–	17.1	–
LRF 766	Partial dentary with dentition	*Weewarrasaurus pobeni*	Holden’s	4.7	3.2	–	–
LRF 3076	Partial dentary with dentition	*Weewarrasaurus pobeni* (holotype)	Wee Warra	6.8	7.6	–	–

**Notes:**

Measurements are in millimetres.

CH, crown apicobasal height; CL, crown mesiodistal length.

The electronic version of this article in portable document format will represent a published work according to the International Commission on Zoological Nomenclature (ICZN), and hence the new names contained in the electronic version are effectively published under that Code from the electronic edition alone. This published work and the nomenclatural acts it contains have been registered in ZooBank, the online registration system for the ICZN. The ZooBank LSIDs (Life Science Identifiers) can be resolved and the associated information viewed through any standard web browser by appending the LSID to the prefix http://zoobank.org/. The LSID for this publication is: urn:lsid:zoobank.org:pub:E8AFFE1D-FACA-4A69-BAA9-5FF6EE1F2EAD. The online version of this work is archived and available from the following digital repositories: PeerJ, PubMed Central and CLOCKSS.

### Phylogenetic analysis

The phylogenetic positions of *Weewarrasaurus pobeni* gen et sp. nov. and an unpublished Lightning Ridge iguanodontian (LRF 3050) were assessed within a recently published ornithischian matrix of Boyd (2015; including modifications by Madzia, Boyd & Mazuch, 2018) that broadly samples Neornithischia (File S1). The characters were equally weighted and analysed in TNT (version 1.5; Goloboff & Catalano, 2016). Initial searching was performed with the ‘New Technology’ search option, using random sectorial searches and the default setting of five rounds of tree fusing, 30 iterations of drifting and 50 iterations of ratcheting. The resulting most parsimonious trees (MPTs) were then subjected to branchbreaking to more fully explore the tree space and identify additional MPTs. Branches with no possible support were collapsed (i.e. ‘rule 3’ of TNT). All characters were unordered, as in the original analysis.

### Dental terminology

**Figure 2:**
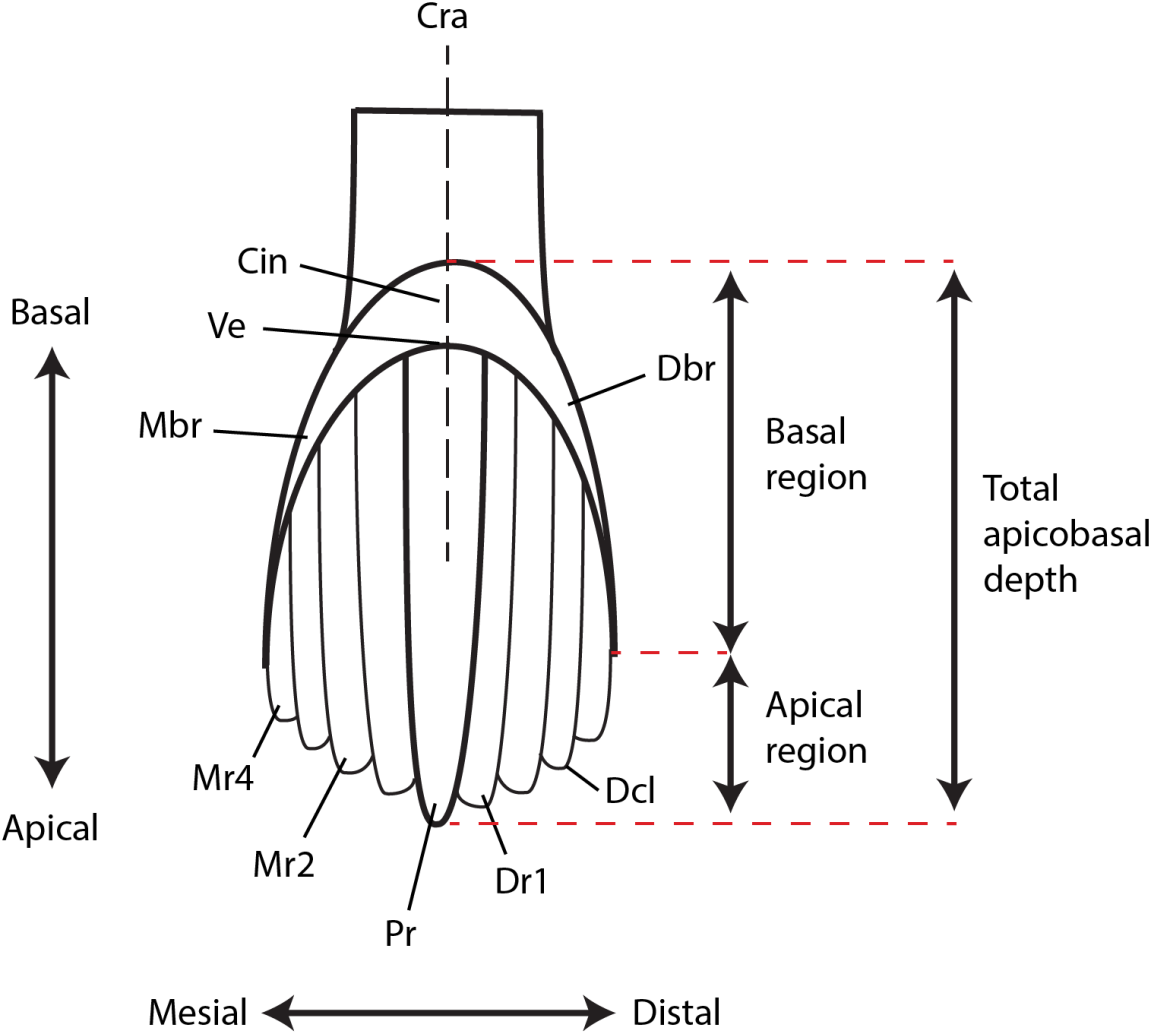
Ornithopod dental terminology. Abbreviations: Cin, cingulum; Cra, central root axis; Dbr, distal bounding ridge; Dcl, denticle; Dr, distal secondary ridge; Mbr, mesial bounding ridge; Mr, mesial secondary ridge; Pr, primary ridge; Ve, vertex. Redrawn and modified from Herne (2014).

For dental terminology, we follow the widely-used terminology of Norman et al. (2004c) with some alterations or additions as proposed by Herne (2014) and outlined here (Fig. 2): Mesial and distal bounding ridges are ridge-like extensions of the cingulum that form the mesial and distal margins of the crown. The vertex refers to the confluence of the mesial and distal bounding ridges. The shape of the vertex is variable and can be U-shaped, V-shaped, W-shaped, or horizontal/linear.

The secondary ridges are numbered successively away from the primary ridge in both mesial (m) and distal (d) directions. Therefore, the secondary ridge immediately adjacent to the primary ridge on the mesial side is denoted Mr1, whereas the fourth secondary ridge on the distal side of the primary ridge is denoted as Dr4.

The crown can be divided into a basal and an apical region. The basal region refers to the area from the cingular vertex to a line drawn between the apical tips of the mesial/distal bounding ridges. The apical region therefore refers to the area apical to the basal region. The central root axis describes a vertical (apicobasal) line drawn through the central axis of the root.

## Geological Setting

The GCF (Rolling Downs Group of the Surat Basin) is a sedimentary unit up to 400 m thick that crops out between the towns of Lightning Ridge (New South Wales) and Surat (Queensland) to the north (Reiser, 1970; Exon, 1976; Green et al., 1997; Price, 1997; Fig. 1A). Sediments of the GCF consist of thinly-bedded and interlaminated fine- to medium-grained sandstone, siltstone, mudstone, and minor coal. Based on sedimentological, palynological, and palaeontological data, Exon (1976) and Green et al. (1997) interpreted these deposits as a relatively complex succession of initially regressive beach or nearshore marine deposits, followed by paralic to deltaic conditions, and finally floodplain deposits in the upper part of the sequence, that accumulated at or near the southeastern margin of the eperic Eromanga Sea.

In the Lightning Ridge district, the GCF is divided into two informal members, the Wallangulla Sandstone member and the overlying Coocoran Claystone member (Moore, 2002) that were deposited at a palaeolatitude of ∼60°S (Hay et al., 1999). The formation is largely obscured on the surface by Quaternary gravels but has been widely explored by opal miners who tunnel into the underlying mid-Cretaceous rocks in search of opal, which occurs in seam and nodular form and also replaces fossils. Preservation of internal structure is variable in these fossils. Some preserve fine details of bone microstructure (e.g. LRF 3067), although most are pseudomorphs. In no case, however, is the subtle distinction between enamel and dentine preserved. Fossil discoveries are almost exclusively a result of the mining process, which unfortunately results in the breakage and/or disassociation of remains and the loss of detailed stratigraphic information.

The GCF is usually considered latest Albian (Raza, Hill & Korsch, 2009); however, new radiometric dates obtained from a reworked ash horizon close to where the specimens described here were found indicate an early Cenomanian age for the GCF at Lightning Ridge (Bell et al., 2019). Exposures of the GCF at Lightning Ridge (and, hence the fossils described here) are therefore younger than the Eumeralla Formation (late Hauterivian–Albian) in Victoria to the south and temporally straddled by the Winton Formation (late Albian–Turonian) in Queensland to the north (Fig. 1C).

## Systematic Palaeontology

Dinosauria Owen, 1842
Ornithischia Seeley, 1888
Clypeodonta Norman, 2014 (sensu Madzia, Boyd & Mazuch, 2018)
Cerapoda Sereno, 1986
Ornithopoda Marsh, 1881
*Weewarrasaurus* gen. nov. urn:lsid:zoobank.org:act:BD3BF258-DBC2-4D37-8143-07C24BA8850C.

**Etymology:** Genus, *Weewarra*, from Wee Warra, the locality from which the holotype specimen derives, and *‘saurus’* (Greek), meaning lizard.

**Diagnosis:** Non-iguanodontian ornithopod characterised by the following unique combination of characters: an elongate dentary ramus with slot-like neurovascular foramina on the lateral surface (ratio of posterior-most foramen length/height ≅ 5.0); large dentary tooth crowns (ratio of apicobasal height of largest posterior crown/total dentary height measured at the same alveolus ≅ 0.5); closely-butting secondary apicobasal ridges with a ‘fan-like’ arrangement on the lingual crown surface mesial and distal to the primary ridge; and weakly-developed apicobasal ridges on the labial crown surface.

*Weewarrasaurus* can be further differentiated from *Q. intrepidus* (the only Australian non-iguanodontian ornithopod with an unambiguously assigned dentary) by: ventral margin of dentary sinuous rather than convex; lateral dental parapet is dorsoventrally shallow and convex, rather than dorsoventrally deep, and concave; neurovascular foramina laterally on dentary anteroposteriorly elongate rather than ovate; lateral neurovascular foramina located two-thirds of the distance from the ventral margin of the dentary to the alveolar margin, rather than midway between the margins; viewed medially, dorsoventral height of the dentary ramus (i.e. region ventral to the medial dental parapet) near the posterior end of the tooth row, roughly equals apicobasal tooth crown height, rather than three times crown height; viewed medially, dorsal margin of the dentary ramus shallowly concave, rather than deeply concave; dentary crowns of adjacent tooth families loosely abutting, rather than closely abutting; largest dentary crowns mesiodistally broader than apicobasally tall (ratio of mesiodistal width/apicobasal height ∼1.4, rather than ∼0.75); and primary ridge nearer middle of central crown axis rather than more distally offset.

**Figure 3:**
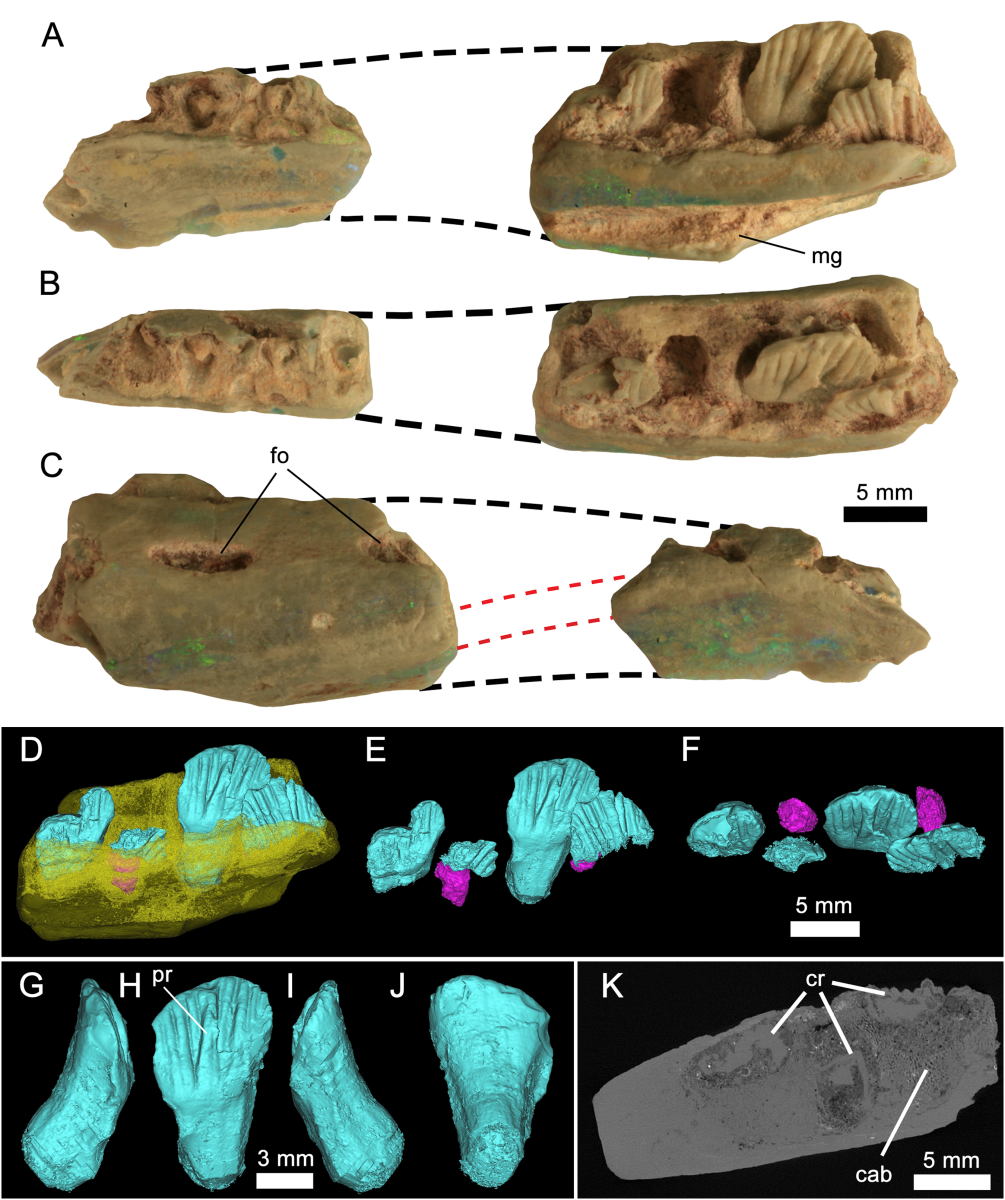
*Weewarrasaurus pobeni* gen. et sp. nov. (LRF 3067; holotype). Right dentary in (A), medial; (B), dorsal; and (C) lateral views. Dashed black lines represent approximate contours of the missing areas. Dashed red lines indicate the distinctive banding pattern in the opal used to estimate the extent of the missing area. (D–F) Three-dimensional renders of the posterior dentary fragment in (D) lingual view showing erupted (blue) and developing germ teeth (pink); (E) Same as (D) but with dentary removed; (F) dorsal (occlusal) view of tooth row. (G-J) Three-dimensional render of the best-preserved tooth in (G) mesial, (H) lingual, (I) distal, and (J) labial views. (K) MicroCT scan of the posterior dentary fragment in axial view showing preservation of cancellous bone. Abbreviations: cab, cancellous bone; cr, tooth crown. Photo credit: Phil Bell.

**Figure 4:**
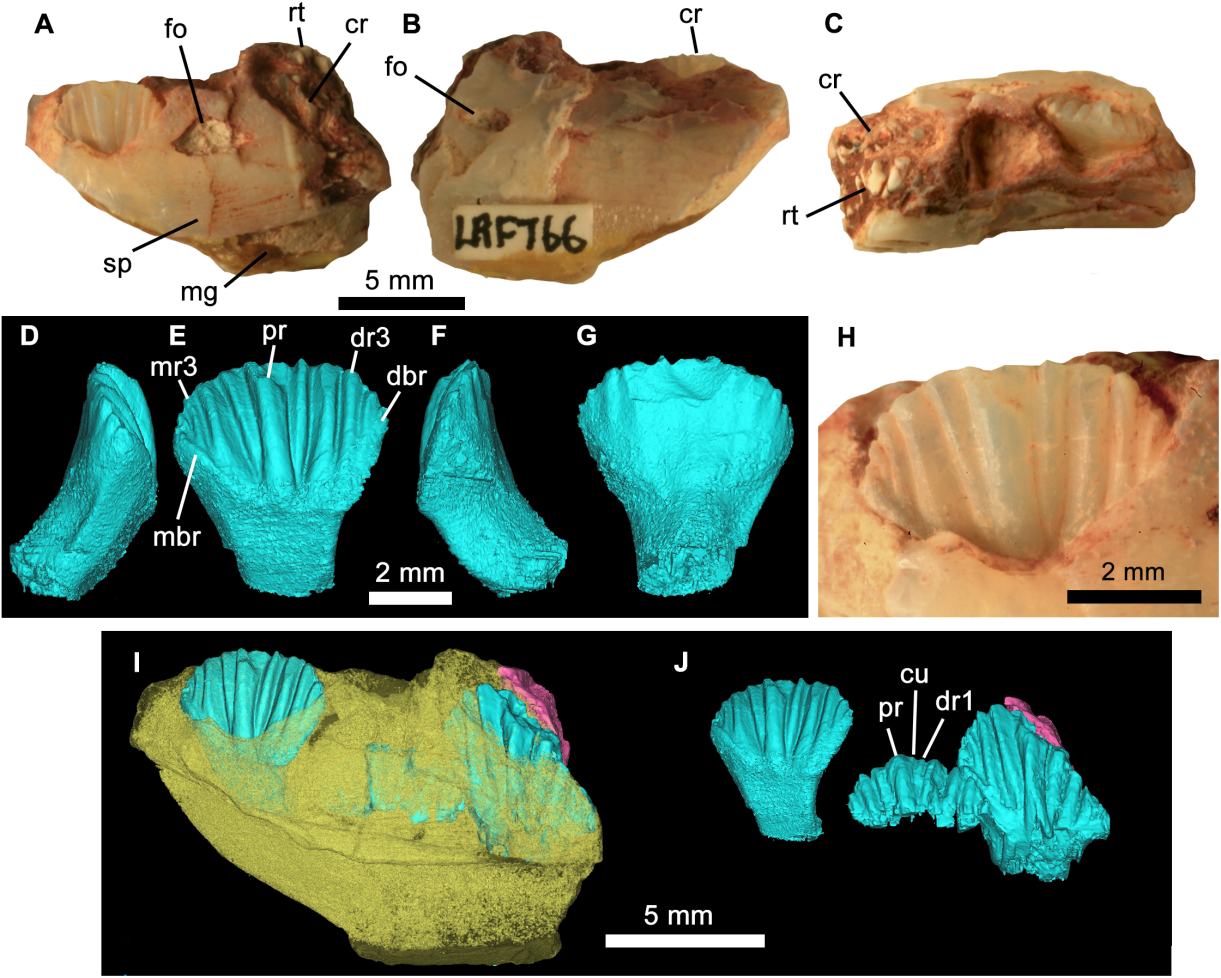
*Weewarrasaurus pobeni* gen. et sp. nov.(LRF 766). Right dentary in (A) medial, (B) lateral, and (C) dorsal views. Three-dimensional render of the erupted crown and root in (D) mesial, (E) lingual, (F), distal, and (G) labial views. (H) Close up of erupted crown. (I and J) Three-dimensional renders of the dentary (yellow) showing erupted and germ teeth (blue) and exposed root (pink) in lingual view. (J) Same view as (I) but with dentary removed. Abbreviations: cr, tooth crown; cu, cusplet; dbr, distal bounding ridge; dr, distal ridge; fo, nutrient foramen; mbr, mesial bounding ridge; mg, meckelian groove; mr, mesial ridge; pr, primary ridge; rt, exposed tooth root; sp, sutural surface for splenial. Photo credit: Phil Bell.

*W. pobeni* sp. nov. urn:lsid:zoobank.org:act:4CA9B960-AD74-4748-B22D-DDEF6BF2498C.

(Figs. 3–4; Table 1)

**Locality and horizon:** The holotype comes from an underground mine at the Wee Warra locality close to the Grawin/Glengarry opal fields approximately 40 km southwest of Lightning Ridge, central-northern New South Wales (Fig. 1A). Fossils derive from the claystone lenses (informally referred to as the ‘Finch clay facies’) within the Wallangulla Sandstone of the GCF (Surat Basin, early Cenomanian; Bell et al., 2019).

**Holotype:** LRF 3067, partial right dentary with dentition.

**Etymology:** In recognition of Mike Poben who acquired and donated the holotype.

**Diagnosis:** As for genus.

**Preservation of LRF 3067:** The holotype dentary was found and broken during routine mining and recovered as two separate and non-contiguous pieces. In addition to their size and overall morphology, they unequivocally belong to a single element based on their preservation in both common (‘potch’) and precious opal (SiO_2_−*n*H_2_O). In particular, successive bands of predominantly green–blue precious opal and blue–grey potch, visible in both cross-section and on the lateral surfaces (Fig. 3C), can be matched on both pieces that attest to their former connection. Such patterns and colours in opal are unique to each piece and will not be replicated in other specimens owing to the peculiarities in the amorphous silica spheres that are responsible for such features (e.g. Sanders, 1964; Sanders & Murray, 1978). In the same way, the orientation of this distinctive banding pattern was used as the basis for estimating the amount of missing material between the two pieces (Figs. 3A–3C).

**Referred materials:** LRF 766, partial right dentary with dentition from the Holden’s locality on the Three Mile opal field immediately south of the Lightning Ridge town boundary (Fig. 1A). It is referred to *W. pobeni* based on shared characteristics of the dentition, including dentary teeth that have a mesiodistal length at least 0.57 times the dorsoventral height of the dentary (see Description).

## Description

### Dentary

The following descriptions are based on the holotype (LRF 3067) except where noted. LRF 3067 is an incomplete right dentary comprising two non-contiguous pieces (Fig. 3). Based on the angle of attenuation between the medial and lateral sides of the jaw and the distinctive colour bands of opal, it is estimated that about the middle third of the dentary is missing. Both fragments are broken rostrally and caudally, therefore nothing is preserved of either the coronoid process or the symphysis, although the ventral margins (exclusive of the symphysis) of both pieces appear to be intact. The rostral fragment is 20 mm long and retains parts of at least five (possibly six) alveoli, four of which contain broken roots. The caudal section of the dentary measures 25 mm long and preserves three alveoli and remnants of a fourth at its caudal end, making a total of eight or ten alveoli, although the total number of alveoli was certainly higher taking into account the missing portion of the dentary. The total number of alveoli is estimated to have been ∼12. In the caudal section, tooth crowns are preserved in all four alveoli. The only complete tooth, however, is preserved in the penultimate alveolus.

Taken together, the ventral and alveolar margins of the dentary would have been anteriorly convergent. The medial surface is flat and, in the rostral fragment, meets ventrally with the lateral surface at an acute angle, conveying a V-shaped sagittal cross-section. The abraded medial surface obscures any detail of the contact with the postdentary bones; however, in LRF 766, the medial wall of the dentary is ornamented across its ventral one-third by a number of fine, rostrocaudally-oriented striae that likely formed the contact for the lateral surface of the splenial (Fig. 4A). Just dorsal to this ornamented region in LRF 766, and in line with the third preserved alveolus, is a large triangular opening, which we interpret as a replacement foramen (‘special foramina’ of Edmund, 1957), as is characteristic of all genasaurians (Norman et al., 2004c).

Rostrally, the dentary is weakly deflected rostromedially where it would have extended to form the symphysis; however, the symphysis itself is missing. The ventral margin is sinuous. Rostrally, the margin is procurved towards the symphyseal margin, as in *Thescelosaurus neglectus* (Galton, 1997, Fig. 1), whereas the caudal part of the margin, although broken on the holotype, appears to have been ventrally convex. The lateral surface is dorsoventrally convex; the inflection point of that curvature occurs at a point equal to two-thirds the height of the dentary. This inflection point forms a low (buccal) ridge on the caudal dentary fragment. This ridge is confluent with the ventral margin of a large slit-like foramen that pierces the lateral dentary wall. Similar large, slit-like foramina are present in *Changchunsaurus parvus* (Jin et al., 2010). A second incompletely-preserved foramen is exposed at the broken rostral end of the dentary fragment. Although the jaw is opalised, micro-CT scans clearly reveal spongy cancellous bone internally, which is highly unusual for this mode of preservation (Fig. 3H).

The mandibular canal is straight, and open ventromedially. It is deepest caudally and rostrally tapering, remaining open until it reaches a point in line with the first preserved alveolus at which point the ventromedial surface is broken, obscuring further details of this structure.

### Dentary teeth

The teeth are loosely abutting, lacking the tightly interlocking arrangement present in *Muttaburrasaurus* spp., *Qantassaurus intrepidus* and more derived Iguanodontia (Zheng et al., 2014). Micro-CT imagery confirms the presence of a single developing germ tooth—typical of most ornithischians except derived Hadrosauriformes and Ceratopsidae (Norman, 2004; Dodson, Forster & Sampson, 2004)—that emerges lingually to the active tooth (Fig. 3F). In lingual view, the tooth crowns are basally deep (the basal region being at least 1.5 times the height of the apical region) and have a low parabolic apical margin (Fig. 3G). Unworn crowns are mesiodistally longer than high in LRF 766, but are higher than mesiodistally long in LRF 3067 (Table 1). Relative to the height of the dentary (measured laterally), the tooth crowns are unusually large, having a mesiodistal length 0.57 times the dorsoventral height of the dentary. In contrast, this same ratio is 0.31–0.36 in *Q. intrepidus*, depending on the position along the tooth row. Lingually, the prominent primary ridge is centrally located and mesiodistally ‘pinched’ at its mid-height and therefore roughly hour-glass shaped (Figs. 3G and 4D). In LRF 3067, there are between four and five mesial secondary ridges, and five or six distal secondary ridges, which are constrained by bounding ridges and conveying an overall fan-shaped arrangement. The secondary ridge Dr6 forms little more than an apical denticle, and could be referred to as an unsupported denticle. In LRF 766, four secondary ridges are developed both mesially and distally to the primary ridge and unsupported denticles are found at positions Mr4 and Dr4. In LRF 3067, the secondary ridge Mr1 tapers basally, pinching out within the paracingular fossa, whereas Mr2–5 are parallel sided. Distally, Dr1, Dr4, and Dr5 are basally tapering whereas Dr2 and Dr3 are parallel sided. The overall arrangement of primary and secondary ridges is therefore less symmetrical than in LRF 766. Lingually, the mesial and distal bounding ridges descend towards the central root axis and meet with a horizontal basal ridge forming the cingular vertex, which is shaped like an inverted trapezoid. A distinct cingulum is absent. Apicobasal ridges on the labial surface of the crown are present, but poorly developed in LRF 3067. Labially in LRF 766, the apicobasal ridges are shallow and round-crested and merge with the crown surface towards their bases, as in Victorian ornithopod dentary type II (VOD type II of Herne, 2014). The opalised preservation prevents determination of whether the enamel covered both the labial and lingual surfaces or was restricted to the lingual surface of the crown. The root is short (approximately equal in height to the crown), cylindrical and labially curved.

#### Remarks

Norman (2014) diagnosed Clypeodonta based partly on several characters of the dentary teeth: (1) Laterally compressed, asymmetrical and shield shaped in lingual aspect; (2) apicobasally curved (convex) crowns, and; (3) strong primary ridge flanked by a variable number of less prominent secondary ridges. All of these features are present in *W. pobeni* although the mesiodistal asymmetry of the crown is less marked in *W. pobeni* compared to most other ornithopods except *Q. intrepidus* (NMV P199075). Norman (2014) also commented on the asymmetrical distribution of enamel (thicker lingually than it is labially in dentary teeth) in his diagnosis of Ornithopoda; however, these features are not preserved in any of the GCF fossils. *W. pobeni* lacks the tightly packed tooth arrangement typical of derived Iguanodontia but does possess curved roots; a feature of most iguanodontians (unknown in *Q. intrepidus*) but also *Hypsilophodon foxii* (Galton, 1974) and *Parksosaurus warreni* (Boyd, 2014). The mesiodistally broad crowns (i.e. not ‘lozenge-shaped’; *sensu* Scheetz, 1999) together with the presence of apicobasal ridges on the lingual surface of the crown suggest *W. pobeni* probably occupied a position close to the base of Ornithopoda, which is also reflected by the results of our phylogenetic analysis (see Phylogenetic Analysis). The presence of dentary teeth with 10 or more ridges is an unambiguous synapomorphy for the clade containing *Weewarrasaurus* and crownward ornithopods (see Results section). Within Ornithopoda, this feature of the dentary teeth is present in the rhabdodontids *Mochlodon vorosi, Mochlodon suessi*, *Rhabdodon priscus*, *Zalmoxes robustus,* and *Zalmoxes shqiperorum* and *Atlascopcosaurus loadsi*.

Relative to the height of the dentary, the tooth crowns are unusually large, having a mesiodistal length equal to 0.57 times the dorsoventral height of the dentary itself. By contrast, dentary height in *Q. intrepidus* varies from 2.6–3.0 times crown length depending on the position along the tooth row. Dentaries of *Atlascopcosaurus loadsi* and *L. amicagraphica* are unknown (Herne, 2014). An isolated dentary, NMV P182967, from the Eumeralla Formation also differs in having proportionately small teeth (dorsoventral height of the dentary/mesiodistal crown length = 2.9). Several other taxa possess maxillary and/or dentary teeth that are greatly enlarged relative to the tooth bearing element (e.g. *Matheronodon provincialis*, Godefroit et al., 2017; *Lanzhousaurus magnidens*, You, Ji & Li, 2005); however, such cases appear to be restricted to Iguanodontia.

*Weewarrasaurus pobeni* can be distinguished from *Q. intrepidus*—the only named basal neornithischian from Australia with an unequivocally assignable dentary—in having a relatively shallow dentary, basally shallow crowns with a centrally located primary ridge and basally-tapering primary and (some) secondary ridges. The sinuous ventral margin of the dentary also differs from *Q. intrepidus*, where the margin is ventrally convex over its length (Rich & Vickers-Rich, 1999, Fig. 8) (Herne et al., in press). Labial ridges are present on the crowns of *W. pobeni, Q. intrepidus* and the single known crown of *Kangnasaurus coetzeei*, but those of *W. pobeni* differ in being weakly developed, not extending the full height of the crown surface, and having blunter crests. The labial ridges on the crowns of VOD type III (Herne, 2014, Fig. 6.10) appear more strongly developed than those of *W. pobeni*; however, the basal region of the crowns cannot be observed for comparisons. Round-crested labial ridges are more strongly developed in *P. warreni* and *Thescelosaurus neglectus* (Galton, 1997, Fig. 2), than in *W. pobeni*. In contrast to *W. pobeni*, the labial surfaces of the crowns of *Hypsilophodon foxii* (Galton, 1974), VOD type IV (Herne, 2014), and most basal iguanodontians (Norman, 2004) are smooth. It is of note that VOD type IV (NMV P182967) was previously referred to *Atlascopcosaurus loadsi* (Rich & Vickers-Rich, 1989); however, past referrals of all dentary material to that taxon are not presently considered as valid (Herne, 2014).

Ornithopoda indet. A

**Materials:** AM F105667, AM F106297, incomplete right dentaries with dentition.

**Locality and horizon:** AMF105667 collected by Ed Long at Dead Bird (locality), Coocoran opal field; AMF106297 mined by Marcel Miltenberg at Emu’s (locality), Coocoran opal field (Fig. 1A). Both Wallangulla Sandstone, GCF, Cenomanian, Rolling Downs Group of the Surat Basin.

**Description**

**Figure 5:**
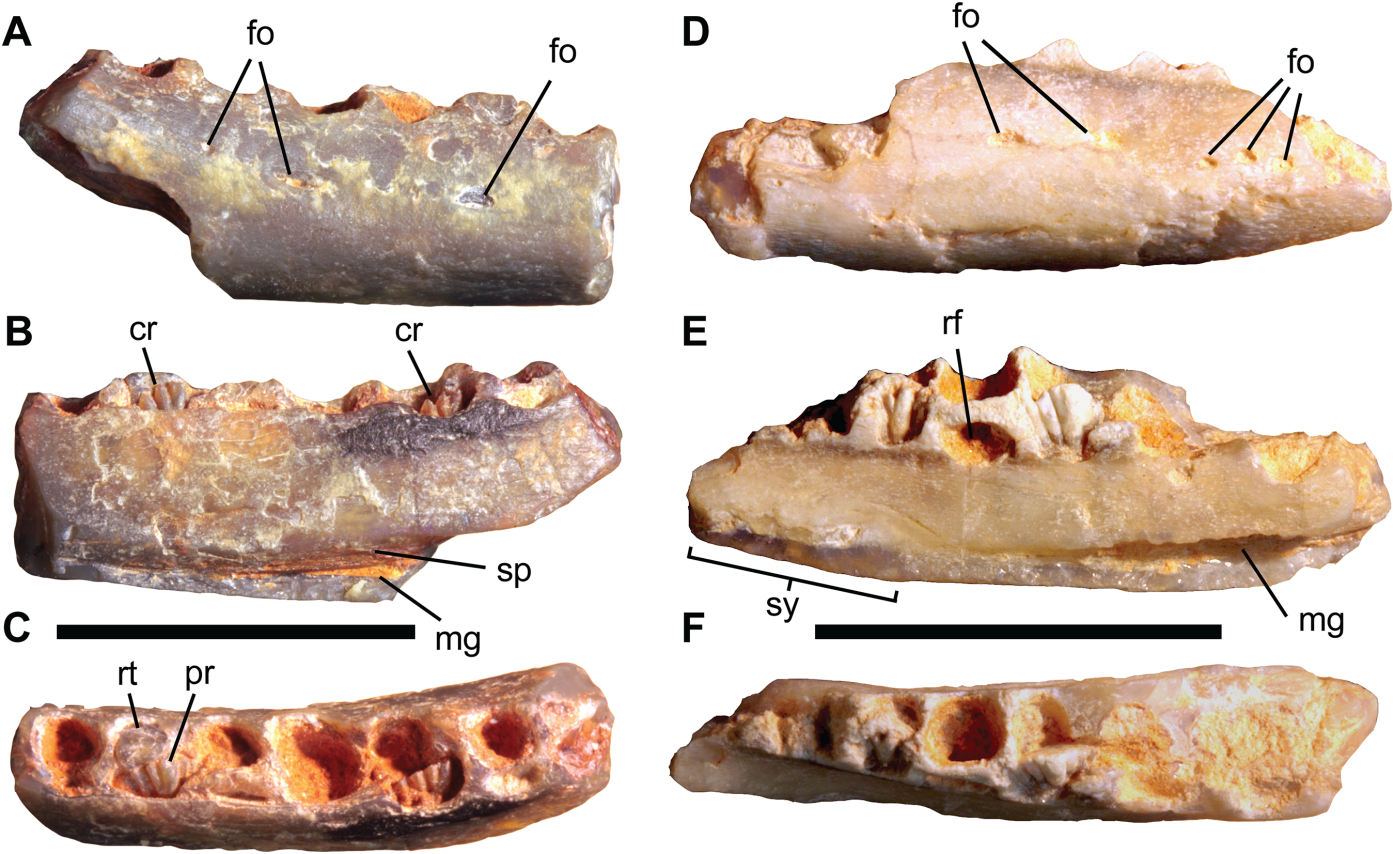
Isolated ornithopod dentaries from the Griman Creek Formation. Right dentary (AM F106297) in (A) lateral, (B) medial, and (C) dorsal views. Right dentary (AM F105667) in (D) lateral, (E) medial, and (F) dorsal views. Scale bars equal 10 mm. Abbreviations: cr, crowns of germ teeth; fo, foramen; mg, Meckelian groove; pr, primary apicobasal ridge; rf, replacement foramen; rt, broken tooth root; sp, sutural contact for the splenial; sy, symphyseal region. Photo credit: Matt Herne.

AM F105667 and AM F106297 are the smallest but among the most completely-preserved ornithopod dentaries from the GCF (Fig. 5; Table 1). The following description is based on the more complete specimen, AM F105667 (Figs. 5D–5F), except where noted, although both appear indistinguishable based on the available material. Viewed dorsally, the dentary is gently bowed (concave laterally) and anteriorly tapering in lateral view, the latter condition being plesiomorphic for Ornithopoda (Boyd, 2014). The coronoid process is not preserved, although curvature of the caudal part of the tooth row (concave in medial view) and the presence of a suture for the splenial (visible in AM F106297), suggest that the break at the caudal end was close to the coronoid process. The alveolar margin, which is damaged at both ends, preserves at least eight alveoli, although the transverse partitions between them in the rostral and caudal-most parts of the tooth row are difficult to discern, making this number tentative. As the caudal end of the dentary is missing, additional alveoli were likely present caudally. The lateral surface is dorsoventrally convex and pierced by two elliptical foramina (three in AM F106297) at about two-thirds the height of the dentary, in line with alveoli four and five, respectively. More rostrally, three or four smaller foramina occur in a roughly horizontal line in the region of the predentary contact, as occurs in most basal neornithischians (Norman, Witmer & Weishampel, 2004a; Norman et al., 2004c). Laterally, the dental parapet is dorsoventrally low and forms a step where it merges with the buccal shelf, which is dorsally convex over its length. Viewed laterally, the dorsally convex buccal ridge and ventrally convex ventral margin converge anteriorly, giving the rostral part of the dentary a ‘bullet-like’ appearance. The convex ventral margin of the dentary is similar to *Q. intrepidus* (NMV P199075) and *Gasparinisaura cincosaltensis* (MUCPv-208); however, given that the caudal end of AM F105667 is missing, the overall shape of the complete dentary is uncertain. The low, lateral dental parapet differs from the dorsoventrally deep dental parapet of *Q. intrepidus*, the buccal shelf of which also differs from AM F105667 in being dorsolaterally concave. In dorsal aspect, the medial and lateral margins of the tooth row converge rostrally and the alveoli are simultaneously reduced in size. Whether or not a diastema was present and the form of contact with the predentary are unknown. Medially, the rostroventral region of the dentary is dorsoventrally concave, scooping medially where it would have contacted the adjoining dentary at the symphysis. The symphysis itself is abraded and likely incomplete but forms a line parallel with the long axis of the dentary in dorsal view. Due to narrowing of the rostral part of the tooth row, medial inflection of the symphysis does not extend beyond the medial margin of the main body of the dentary. The Meckelian groove extends caudally along the ventromedial edge of the dentary from the caudal end of the symphyseal margin (corresponding roughly to the juncture of alveoli three and four). The Meckelian groove expands into the triangular meckelian canal. The caudal end of the Meckelian canal is not preserved on the specimens. The medial surface of the dentary, dorsal to the Meckelian groove and canal, is flat. The presence of a single nutrient foramen per alveolus, as characteristic of all genasaurians (Norman et al., 2004c), is indicated at aveolus four in AM F105667.

The largest and best-exposed crown (position D5) has a parabolic apical margin and a prominent and basally tapering primary ridge on the lingual surface. This basal taper is accentuated by a small ‘cusplet’ on the mesial edge of the primary ridge, similar to LRF 766, which makes it appear apically bifurcated. Two secondary apicobasal ridges are present mesially and distally with respect to the primary ridge, although additional ridges are likely present but are obscured by the surrounding bone. The primary and secondary ridges form a fan-shaped arrangement. The labial surface of the crown appears to be smooth and devoid of apicobasal ridges, similar to *Hypsilophodon foxii* (Galton, 1974), VOD type IV (Herne, 2014), and most basal iguanodontians (Norman, 2004). In AM F106297, the broken roots of teeth are visible in alveoli two and seven (as preserved). The roots are circular in cross section, unlike the D-shaped, or grooved roots, seen in roots of *Tenontosaurus tilletti, R. priscus* and more derived iguanodontians (Scheetz, 1999). CT scans were unfortunately unavailable for either AM F106297 or AM F105667, leaving the remaining dental morphology unknown.

**Remarks**

The shield-like form of the tooth crowns and the presence of a primary ridge surrounded by less prominent secondary ridges is a combination of features found only in Clypeodonta. Similar to *W. pobeni*, AM F105667 has circular roots in cross section and lacks the tightly packed tooth arrangement typical of most Iguanodontia (except the rhabdodontid *Zalmoxes* spp.). AM F106297 and AM F105667 differ from *Q. intrepidus* (NMV P199075) in their more elongate proportions and in having unworn tooth crowns with smoother labial surfaces. In this respect, AM F106297 and AM F105667 more closely resemble VOD type IV (Herne, 2014) and *W. pobeni*. The convex ventral margin on the dentary, the form of the lateral neurovascular foramina, and dorsoventrally-limited contact surface for the splenial of AM F106297 and AM F105667 differs from the procurved ventral margin, elongate foramina, and more expansive splenial sutural surface in *W. pobeni.* We therefore consider AM F106297 and AM F105667 to pertain to a second taxon of ornithopod.

Ornithopoda indet.

**Material:** LRF 660, premaxillary tooth

**Locality and Horizon:** Olga’s (locality) on the Coocoran opal field, New South Wales (Fig. 1A); Wallangulla Sandstone, GCF, Cenomanian, Rolling Downs Group of the Surat Basin.

**Description**

**Figure 6:**
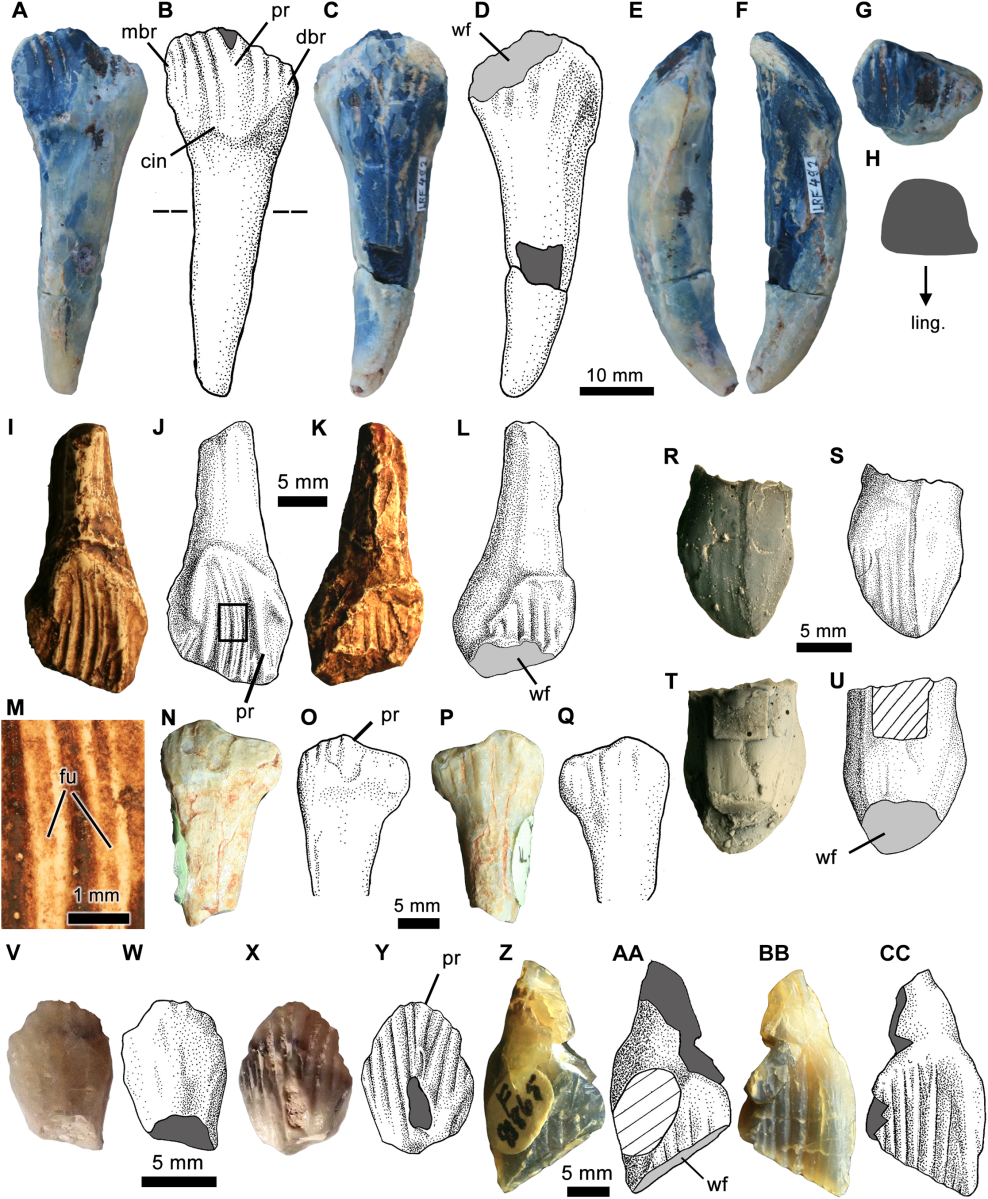
Isolated ornithopod teeth from the Griman Creek Formation. (A, C and E–G) Photographs and (B and D) interpretive illustrations of a complete dentary tooth (LRF 492) in (A and B) lingual, (C and D) labial, (E) mesial, (F) distal, and (G) apical views. (H) Cross-section of the root taken at the level indicated by the dashed line in (B). (I and K) Photographs and (J and L) interpretive illustrations of a nearly complete maxillary tooth (QM F14421; cast of AM F119236) in (I and J) lingual and (K and L) labial views. (M) Close up of secondary ridges denoted by boxed region in (J). (N and P) Photographs and (O and Q) interpretive illustrations of worn dentary tooth (AM F119236; i.e. QM F14420, cast) in (N and O) lingual and (P and Q) labial views. (R and T) Photographs and (S and U) interpretive illustrations of premaxillary tooth (LRF 660; cast) coated in ammonium chloride (for clarity) in (R and S) labial and (T and U) lingual views. (V and X) Photographs and (W and Y) interpretive illustrations of dentary tooth (AM F112862) in (V and W) lingual and (X and Y) labial views. (Z and BB) Photographs and (AA and CC) interpretive illustrations of dentary tooth (AM F112862) in (Z and AA) lingual and (BB and CC) labial views. Dark grey in (B) and (D) indicates broken surfaces. Cross hatching in (U) indicates area obscured by archiving label. Abbreviations: art, casting artefact; for, foramen; fu, furrow on secondary ridge; pr, primary ridge; ling, lingual; wf, wear facet. Photo and illustration credit: Phil Bell.

The isolated crown, LRF 660, is folidont (=lanceolate; Hendrickx, Mateus & Araújo, 2015), mesiodistally as long as it is tall and has a distinct basal constriction (Figs. 6R–6U). A basal constriction is widespread among non-iguanodontian neornithischians as well as the unnamed Vegagete rhabdodontid (Dieudonné et al., 2016), but is absent in most heterodontosaurids (Butler et al., 2009). The crown has a symmetrical ‘D’-shaped cross-section (sensu Hendrickx, Mateus & Araújo, 2015) with a maximum labiolingual width only slightly less than the mesiodistal length. In lingual aspect, the apex of the crown forms a blunt point and is distally offset relative to the central root axis. The mesial margin is convex whereas the distal margin is comparatively straight in labiolingual view, giving the tooth the overall appearance of being weakly recurved. Although the mesial and distal margins form distinct carinae, there are no marginal denticles; however, there are at least three (possibly four) poorly expressed mesial secondary ridges on the labial surface (Figs. 6R and 6S). Marginal denticles are similarly absent in the premaxillary teeth of *Changchunsaurus parvus, Haya griva,* and *Thescelosaurus neglectus* (Jin et al., 2010; Boyd, 2015) as well as in the more basal neornithischian *Yandusaurus hongheensis.* The secondary ridges are parallel to the primary ridge, and become less distinct basally, gradually terminating at a point approximately in line with the mesiodistally longest point of the tooth. The primary ridge is more strongly expressed than the secondary ridges and extends basally onto the root; however, in no case are any of the ridges as prominent as would be expected on maxillary or dentary teeth. The presence of distinct primary and secondary ridges in LRF 660 differs from the premaxillary teeth of *Hypsilophodon foxii* (Galton, 1974) and *Thescelosaurus neglectus* (Boyd, 2014), which have finer, more numerous apicobasal ridges, and also from *Changchunsaurus parvus, Jeholosaurus shangyuanensis*, and *Zephyrosaurus schaffi*, which have smooth crowns (Sues, 1980; Barrett & Han, 2009; Jin et al., 2010). No premaxillary teeth of any other Australian ornithopod are known, although two edentulous premaxillae (NMV P208539, NMV P212800; Herne, 2014) from the Wonthaggi Formation (Victoria) (= ‘undifferentiated Strzelecki Group’ of Toslini, McLoughlin & Drinnan, 1999) preserve alveoli that suggest teeth considerably smaller than LRF 660. Secondary ridges are absent on the distal half of LRF 660 and there is no cingulum. Lingually, there is a large, steep and apicobasally concave wear surface that covers approximately half of the height of the crown. Subsequently, the presence/absence of lingual apicobasal ridges cannot be determined.

**Remarks**

The combination of a basally-constricted, weakly recurved crown with the absence of both marginal denticles and a cingulum identifies LRF 660 as a probable premaxillary tooth. The presence of premaxillary teeth is traditionally regarded as a feature of non-iguanodontian neornithischians; however, premaxillary teeth are present in *Tenontosaurus dossi* (single alveolus, tooth not preserved; Winkler, Murray & Jacobs, 1997) and the Vegagete rhabdodontid (three teeth; Dieudonné et al., 2016). The presence of distinct (albeit weak) primary and secondary ridges in LRF 660 is a feature normally associated with maxillary and dentary teeth of basal iguanodontians although the only preserved premaxillary crown described for any basal iguanodontian (that of the Vegagete rhabdodontid) lacks apicobasal ridges and is unlike LRF 660 and all other ornithopods in its ‘wedge’-like expansion of one (either labial or lingual) side (Dieudonné et al., 2016, p. 10) and the absence of both a cingulum and carinae. Herne (2014) described two edentulous ornithopod premaxillae (NMV P208539, NMV P212800) from the Wonthaggi Formation (Victoria), which likely represent two distinct taxa and have higher tooth counts (three or four in NMV P208539; five in NMV P212800) than would be expected among basal iguanodontians. Although no teeth were present in the Victorian premaxillae, the large size of LRF 660 would appear to preclude it from either of these taxa as well as from the dentaries from the GCF described here. Furthermore, as premaxillary teeth are unknown above Dryomorpha, we consider LRF 660 to represent possibly a third but indeterminate ornithopod taxon from the GCF.

Ornithopoda indet.

**Materials:** AM F112862, isolated right dentary tooth.

**Locality and horizon:** Allah’s (locality) on the Coocoran opal field, New South Wales (Fig. 1A); Wallangulla Sandstone, GCF, Cenomanian, Rolling Downs Group of the Surat Basin.

**Description**

AM F112862 is a small, unworn crown (Figs. 6V–6Y). The basal part of the primary ridge is damaged but the tooth is otherwise well preserved. In lingual view (Fig. 6X), the crown is roughly diamond shaped, symmetrical (the basal region being subequal in height to the apical region) with a steep parabolic apical margin, contrasting with the low apical margin in *W. pobeni* (LRF 766, LRF 3067). There is no apical wear facet, which indicates that these contours are real and not the result of wear. The primary ridge is centrally located and appears to taper basally although damage in this region makes the latter observation equivocal. There are four parallel secondary ridges constrained by bounding ridges that diverge apically from either side of the primary ridge. Given the overall mesiodistal symmetry of the crown, it is not possible to distinguish distal from mesial. Dr1–2 and Mr1–2 converge near the base of the primary ridge whereas Mr1 and Dr1 only extend a short distance from the apex (i.e. they do not reach the base of the primary ridge). There is no basal cingulum. The labial surface of the crown is smooth and lacks apicobasal ridges, similar to *W. pobeni*, *Hypsilophodon foxii* (Galton, 1974), VOD type IV (Herne, 2014), and most basal iguanodontians (Norman, 2004).

**Remarks**

Mesiodistally symmetric crowns with a centrally placed and modestly pronounced primary lingual ridge and a number of secondary ridges, as seen in AM F112862, are characteristic of non-iguanodontian ornithopods (Scheetz, 1999), including *W. pobeni* and *Q. intrepidus* (NMV P199075). In more derived iguanodontians (e.g. *Muttaburrasaurus langdoni, Tenontosaurus tilletti, Zalmoxes* spp.), the primary ridge becomes more strongly developed and, to some extent, offset from the midline. The absence of labial ridges in AM F112862 is a typical dryomorphan feature (convergently absent in *Hypsilophodon foxii* (Galton, 1974)) but is also found in *W. pobeni*, AM F105667 and an undescribed dentary from the Eumeralla Formation in Victoria (NMV P182967; dentary Type IV of Herne, 2014). We therefore assign LRF 766 to an indeterminate non-dryomorphan ornithopod.

Although AM F112862 shares with *W. pobeni* the aforementioned features, it can be differentiated from the latter by its more diamond-shaped and apicobasally tall lingual face. All three specimens (AM F112862, LRF 766, LRF 3067) bear different numbers of secondary lingual ridges; however, this feature is known to be ontogenetically variable in related taxa (e.g. *Talenkauen santacrucensis*; Egerton et al., 2013). The teeth in the two partial dentaries, AM F105667 and AM F106297, are not adequately exposed to permit most comparisons, although AM F105667 shares with AM F112862 the absence of apicobasal ridges on the labial crown surface. This feature is also shared with VOD type IV (NMV P182967, Herne, 2014), although AM F112862 differs in having fewer secondary ridges. The absence of labial apicobasal ridges also differentiates AM F112862 from *Q. intrepidus*. Although AM F112862 can be differentiated from *W. pobeni,* we cannot reject the possibility that AM F112862 is from the same taxon as AM F105667 and AM F106297.

Iguanodontia Dollo 1888
Iguanodontia indet.

**Material:** AM F119282 (QMF 14421, a cast), maxillary tooth.

**Locality and horizon:** McNamara’s (locality) on the Three Mile opal field, Lightning Ridge, New South Wales (Fig. 1B); Cenomanian, GCF, Rolling Downs Group in the Surat Basin.

**Description**

Molnar (1996) figured and briefly described AM F119282 (referring to the cast, QMF 14421), which consists of the crown and incomplete root of a relatively large left maxillary tooth (Figs. 6I–6L). The crown is spatulate but strongly asymmetrical, in part resulting from an oblique wear facet on the occlusal margin. In addition, the asymmetry of the crown results from: a mesially offset, ‘V’-shaped cingular vertex relative to the central root axis, both labially and lingually; and viewed lingually, distal offset of the primary ridge and the obliquely sloping distal bounding ridge at roughly 45° to the central root axis. The mesial bounding ridge is subvertical. The distally offset primary ridge is oriented sub-parallel to the central root axis and forms the apical-most point on the crown (Fig. 6J). Mesial to the primary ridge, there are five secondary ridges, which is similar to *Atlascopcosaurus loadsi* (NMV P166409), greater than *L. amicagraphica* and *Leaellynasaura* sp. (NMV P186440, Herne, 2014) but only about half as many as *Muttaburrasaurus* sp. (QM F12541). The mesial secondary ridges are curved with the concave margin of the curve facing mesially such that they form a fan like arrangement, similar to *Atlascopcosaurus loadsi* (NMV P166409, NMV P157390). Each secondary ridge also bears a longitudinal furrow or groove that extends the entire length of the ridge conferring a smooth, ‘m’-shaped cross-section to each ridge (Fig. 6M). Similar longitudinal grooves are visible on some maxillary crowns of *Muttaburrasaurus langdoni* (QM F14921), *Muttaburrasaurus* sp. (QM F12541) and *Leaellynasaura* sp. (NMV P186440) but are otherwise unknown in other neornithischians. As the occlusal edge is worn, it is unknown if each secondary ridge terminated in one or more denticles. Only Mr3 and Mr4 extend to the vertex of the cingulum; Mr1 converges near the base of the primary ridge and Mr5 is truncated by the mesial bounding ridge. Distal to the primary ridge, only a single faint secondary ridge could be discerned, and which sits within a triangular, mesiodistally narrow paracingular fossa.

The lingual surface of the crown has at least five secondary apicobasal ridges, although additional ridges were likely present but are obscured by poor preservation of this surface. The ridges are parallel and extend to the base of the crown. Equally well-developed lingual ridges are present in *Atlascopcosaurus loadsi* (NMV P166409) and *Leaellynasaura* sp. (NMV P229196) although in *L. amicagraphica* (NMV P185991), the ridges are developed only on the apical half of the crown. Lingual ridges cannot be observed on any specimen of *Muttaburrasaurus* due to poor preservation (and/or incomplete preparation) and unequivocal maxillary teeth of *Q. intrepidus* are unknown. The root is basally tapering and elliptical in cross section, being labiolingually wider than it is mesiodistally long. Not enough of the root is preserved to determine whether it was straight or bowed.

**Remarks**

Norman (2014) described asymmetrical maxillary crowns as one of the distinguishing features of Clypeodonta. The reportedly asymmetrical crowns of *Y. hongheensis* and *Zephyrosaurus schaffi* (Norman, 2004) in fact appear to be due to wear on the imbricated crowns. Asymmetry due to a prominent, distally-offset primary ridge together with a mesially-offset, V-shaped cingular vertex, however, is a combination of features seen only in Australian (*Atlascopcosaurus loadsi, L. amicagraphica, Muttaburrasaurus langdoni*) and some Argentinean (*G. cincosaltensis, Talenkauen santacrucensis*; Cambiaso, 2007, Fig. 17) taxa (P. Bell, 2018, M. Herne, 2012, personal observation). Lingual ridges (confirming the presence of enamel), such as those in AM F119282, are absent in more advanced iguanodontians (Norman, 2004). The single prominent (primary) ridge on the labial surface of AM F119282 differs from the two prominent ridges in the maxillary teeth of *Hypsilophodon foxii* (Galton, 2009) and a tooth referred to an unnamed Spanish rhabdodontid (Dieudonné et al., 2016).

Among Australian taxa, AM F119282 is most similar to *Muttaburrasaurus* sp. (QM F12541, QM F14921) in the combination of a strongly asymmetrical crown, distally offset primary ridge, mesially offset cingular vertex and the presence of slender ‘grooved’ secondary ridges. As previously noted by Molnar (1996), AM F119282 differs from *Muttaburrasaurus* in its smaller size and having only about half as many mesial secondary ridges. These differences led Molnar (1996, p. 639) to identify AM F119282 as belonging to a ‘more plesiomorphic species of *Muttaburrasaurus*’. Although the generic referral to *Muttaburrasaurus* is problematic, we agree that AM F119282 represents a basal iguanodontian based on the asymmetrical crown, prominent primary ridge, and well-developed secondary ridges on both labial and lingual surfaces. The discovery of AM F119282 and AM F119236 (a dentary tooth; see below) from the same mineral claim at the McNamara’s locality on the Three Mile opal field, together with the overall rarity of large ornithopod teeth from the GCF in general, lends circumstantial support to the idea that they belong to the same taxon.

Iguanodontia indet.

**Material:** AM F81865, maxillary tooth.

**Locality and horizon:** Mineral claim known as ‘The Boneyard’ at the Nine Mile opal field, New South Wales (Fig. 1B); specimen excavated in mid 1980s by Alex Ritchie and Robert Jones (Australian Museum); Wallangulla Sandstone, GCF, Cenomanian, Rolling Downs Group of the Surat Basin.

**Description**

A left maxillary tooth, AM F81865 (Figs. 6Z–CC), is the largest of the maxillary crowns mentioned (but not described) by Molnar (1996). In general, it is similar in morphology to AM F119282: the crown is spatulate and strongly asymmetrical in labial view, due to the mesially offset cingular vertex, distally offset primary ridge and the steeply angled wear facet. The primary ridge is parallel to the central root axis and forms the apical-most point on the crown (Fig. 6BB). There are seven mesial secondary ridges, two greater than AM F119282 (described above), but similar to *Muttaburrasaurus* sp. (QM F14921), although only about half as many as another referred specimen of *Muttaburrasaurus* sp. (QM F12541). The mesial secondary ridges diverge from the primary ridge but, unlike AM F119282, they are straight rather than forming a fan-like arrangement and longitudinal grooves are restricted to the basal parts of the ridges (Fig. 6CC). Mr1 and Mr2 are truncated by the primary ridge, whereas Mr3 and Mr4 extend to the vertex of the cingulum. A single distal secondary ridge is present within narrow distal paracingular fossa although it does not extend basally to contact the cingulum. The distal bounding ridge is subvertical and parallel to the primary ridge whereas the mesial bounding ridge is oblique, resulting in an acute, ‘V’-shaped cingulum.

The lingual surface of the crown has at least five secondary apicobasal ridges, similar to AM F119282, *Atlascopcosaurus loadsi* (NMV P166409) and *Leaellynasaura* sp. (NMV P229196). These ridges are parallel to the crown-root axis. Not enough of the root is preserved to determine whether it was straight or bowed.

**Remarks**

In most respects, AM F81865 closely resembles AM F119282 (described above). Some minor variation occurs in the number and curvature of the secondary ridges as well as the extent of longitudinal grooves and steepness of the wear facet, all of which we regard as potentially attributable to individual or positional variation within the tooth row. We therefore assign AM F81865 to an indeterminate basal iguanodontian based on the asymmetrical crown, prominent, distally-offset primary ridge and well-developed secondary ridges on both labial and lingual surfaces. We agree with Molnar’s (1996) suggestion that AM F81865 and AM F119282 likely belong to the same taxon and that they differ substantially from the maxillary teeth of *Muttaburrasaurus* spp. in their smaller size and having only about half as many mesial secondary ridges.

Iguanodontia indet.

**Material:** AM F119236 (QMF 14420, a cast), right dentary tooth.

**Locality and horizon:** McNamara’s (locality) on the Three Mile opal field, Lightning Ridge, New South Wales (Fig. 1B); Cenomanian, GCF, Rolling Downs Group of the Surat Basin.

**Description**

AM F119236 (Figs. 6N–6Q) is a poorly preserved crown and root that was recovered from the same mineral claim as AM F119282 (see above) at the McNamara’s locality on the Three Mile opal field. Molnar (1996) mentioned the cast of this specimen (QMF 14420), which he identified it as a possible maxillary tooth, but did not describe it. Based on comparisons with other better-preserved teeth (see below), we tentatively interpret it as a right dentary tooth and describe it as such here. The root is basally tapering but is truncated by a transverse break. Consequently, not enough of the root is preserved to determine whether it was straight or bowed. At the root-crown junction, the distal margin of the crown expands rapidly and presents a strongly convex distal outline in labial view. The mesial margin is comparatively straight (vertical) as there is no equivalent constriction of the root-crown junction. A distinct cingulum is lacking. The crown is strongly asymmetrical and worn (interpreted as a result of both diagenetic and feeding wear) rendering the original shape of the crown indeterminate. As preserved, it is about twice as broad mesiodistally as it is tall, and labiolingually tapering towards the apex. From the broadest point of the tooth, the mesial and distal margins converge abruptly to form a low, obtuse apex. Mesial to the apex, the apical (occlusal) margin is gently convex in labial view, whereas distal to the apex, the apical margin is concave. The apex, which is centrally located on the crown, is formed by what is presumably the primary ridge; however, as with the secondary ridges, the primary ridge has been worn to a nub, presumably as a result of extensive occlusal wear. The primary ridge is shallow, at this point being no more developed than the secondary ridges. Three mesial secondary ridges are present between the primary ridge and the mesial bounding ridge; however, none of these extend far basally onto the labial face. The region distal to the primary ridge is entirely smooth and devoid of any sign of denticles or secondary ridges. Lingually, there are at least three (but possibly more) broad, but faint apicobasal ridges. The most prominent ridge—the middle of the three visible ridges—lies immediately distal to the apex and all ridges are basally tapering, terminating in line with the crown-root constriction.

**Remarks**

The poor preservation of AM F119236 prevents most meaningful comparisons; however, several observations can still be made. Firstly, the apparent absence of a prominent primary ridge on the labial face of the crown is superficially reminiscent of the condition in non-iguanodontian neornithischians, in which there is no distinction between the primary and secondary ridges. This is, however, likely influenced by the degree of wear on AM F119236 and therefore the systematic relevance of this feature cannot be determined with certainty. Moreover, on a better preserved dentary tooth (LRF 492), the prominence of the primary ridge diminishes basally, closely approximating the condition in AM F119236. The extension of the labial secondary ridges to the base of the crown is similar to a variety of neornithischians (e.g. *Haya griva; Hypsilophodon foxii, P. warreni, Talenkauen santacrucensis*), and all basal iguanodontians (Galton, 1974; Norman, 2004; Makovicky et al., 2011; Boyd, 2014). The presence of lingual and labial ridges is characteristic of all non-dryomorphan iguanodontians, except *Hypsilophodon foxii* (Galton, 1974) and most basal iguanodontians (Norman, 2004) where they are limited to the lingual surface. The distribution of these characters therefore only allows a tentative identification for AM F119236 as an indeterminate non-dryomorphan ornithopod, and likely a basal Iguanodontia.

Iguanodontia indet.

**Material:** LRF 492, right dentary tooth

**Locality and horizon:** Molyneux South (locality) on the Coocoran opal field, New South Wales (Fig. 1B); Wallangulla Sandstone, GCF, Cenomanian, Rolling Downs Group of the Surat Basin.

**Description**

An isolated right dentary tooth, LRF 492, is the largest and most complete ornithopod tooth so far recovered from the GCF (Figs. 6A–6H). The root is basally tapering and bowed ventrolabially, as is typical of basal Iguanodontia (Norman, 2004) but also *Hypsilophodon foxii* (Galton, 1974) and *P. warreni* (Boyd, 2014). The distal surface of the root is flattened, giving the root a distinct ‘D’-shaped cross section (Fig. 6H), similar to the squared-off roots of *Tenontosaurus tilletti* and *R. priscus*, although it lacks the longitudinal groove seen in more derived iguanodontians (Scheetz, 1999). The crown is worn labially but appears to be basally deep, having only a short apical region. The primary ridge is centrally located, at least twice as broad as the secondary ridges, and widens apically where it bifurcates into two subsidiary ridges. This combination of features on the primary ridge is similar to the crowns of *Muttaburrasaurus* sp. (QM F12541, QM F14921), *Q. intrepidus* (NMV P199075) and some undescribed Victorian dentaries (NMV P182967, NMV P221082), but differs from others (e.g. NMV P199135) where the primary ridge tapers apically. Denticles in LRF 492 are, however, largely obliterated by the labial wear facet. Distal to the primary ridge, there are two secondary ridges, about half as many as are present in both *Muttaburrasaurus* sp. (QM F12541, QM F14921) and *Q. intrepidus* (NMV P199075). The distal bounding ridge is oriented at roughly 45° to the central root axis and converges basally with the horizontal cingulum at the base of Dr1. The mesial bounding ridge is subvertical. Each ridge terminates in a single denticle, similar to *Muttaburrasaurus* sp. (QM F12541, QM F14921) and all Victorian neornithischians (e.g. NMV P182967, NMV P221082), but distinct from the unsupported denticles (=mammillae) found in *Zalmoxes* spp. (Weishampel et al., 2003), *Tenontosaurus tilletti* (Thomas, 2015) and more derived iguanodontians (Weishampel et al., 2003). Between the mesial bounding ridge and the primary ridge, there are three parallel-sided secondary ridges, which is less than half the number in *Muttaburrasaurus* sp. (QM F12541). The labial surface is flat apicobasally and convex mesiodistally. This surface is dominated by a large, nearly vertical wear facet, basal to which are three or four broad apicobasal ridges (Fig. 6D). The labial surfaces of the dentary teeth are not well enough exposed in any specimen of *Muttaburrasaurus* to allow comparisons.

**Remarks**

A prominent and centrally-placed primary ridge is found in *Hypsilophodon foxii* (Galton, 1974) and Iguanodontia. On the other hand, well-developed secondary ridges that terminate in a single denticle are not found above Dryomorpha where the secondary ridges are progressively lost and are fewer in number than the denticles (Norman, 2004). We therefore assign LRF 492 to an indeterminate basal iguanodontian.

LRF 492 shares similarities (number of secondary ridges; centrally located, apically divergent and ‘split’ primary ridge; horizontal cingulum) with an isolated dentary tooth (QM F52774) from the Winton Formation (originally reported as a maxillary tooth by Hocknull & Cook (2008)); however, LRF 492 is at least five times as large as QM F52774.

Iguanodontia indet.

**Material:** LRF R1556, left jugal.

**Locality and horizon:** Unknown locality at the Coocoran opal field; Wallangulla Sandstone, GCF, Cenomanian, Rolling Downs Group of the Surat Basin.

**Description**

**Figure 7:**
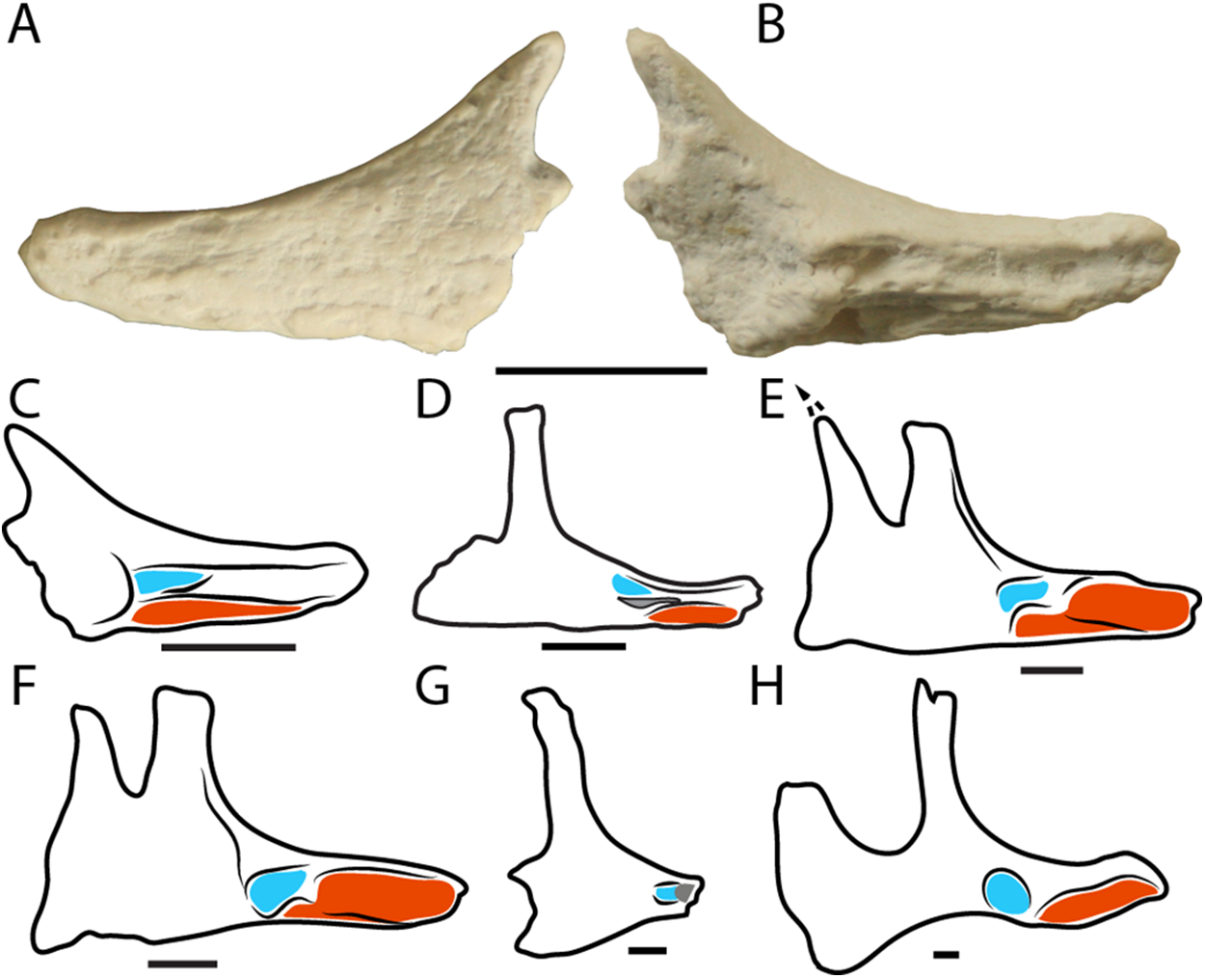
Neornithischian jugals. Left jugal (LRF R1556) in (A) lateral and (B) medial views. (C–H) Schematics of neornithischian jugals in left medial views showing the contact surfaces for the ectopterygoid (blue) and maxilla (red). (C) LRF R1556; (D), *Zephyrosaurus schaffi* (redrawn from Sues, 1980); (E), *Dryosaurus altus* (redrawn from Galton, 1983) (F), *Dysalotosaurus lettowvorbecki* (redrawn from Janensch, 1955) (G), *Thescelosaurus neglectus* (redrawn from Boyd, 2014); (H) *Iguanodon atherfieldensis* (redrawn from Norman, 1986). Scale bars = 1 cm. Scale bars in (E) and (F) are approximate.

LRF R1556 (Fig. 7) is a cast of nearly complete left jugal (the unnumbered original is at the Australian Museum, Sydney). The jugal is a triradiate element with an elongated anterior process, a shorter postorbital process, and an even more abbreviated posterior process although it is possible that the latter is incompletely preserved (see below). The lateral surface of the jugal is dorsoventrally convex as in most ornithopods, and lacks ornamentation (present in *Thescelosaurus*; Boyd et al., 2009) or a boss (present in *Zephyrosaurus schaffi* and *Orodromeus makelai*; Sues, 1980; Scheetz, 1999). The postorbital and anterior processes form an arc delimiting the posteroventral and ventral margins of the orbit, respectively. The anterior process is straight and gently tapering anteriorly, terminating in a bluntly rounded end. This end is imperfectly preserved such that the lacrimal and maxillary contacts are unknown. Medially, a deep groove extends nearly the entire length of the anterior process. This groove is set within a ‘raised’ (i.e. medially expanded) region that demarcates the maxillary process anteriorly and the ectopterygoid contact posteriorly (Fig. 7C). Ventral to this raised region, the ventral surface of the anterior process is dorsoventrally concave where it would have overlapped the posterodorsal surface of the maxilla.

The subconical postorbital process is recumbent and buttressed medially by a ridge that extends from the posterior end of the ectopterygoid contact. The contact surface for the postorbital is not clearly discernible owing to the incomplete preservation of the specimen. The posterior (quadratojugal) process is virtually absent, superficially similar to that of *G. cincosaltensis* (Coria & Salgado, 1996); however, this process is mediolaterally thin and it is possible that part of it is missing. As preserved, the posterodorsally-inclined posterior margin of the posterior process lies essentially ventral to the postorbital process; only a small, posteriorly-directed spur at the base of the postorbital process projects posteriorly beyond the postorbital process. In contrast, the other Australian ornithopods (*L. amicagraphica, Muttaburrasaurus langdoni*) have much longer and dorsoventrally expanded posterior processes (Herne, 2014), although this may be affected by breakage in the current specimen (LRF R1556). Subsequently, only a small portion of the anteroventral margin of the infratemporal fenestra (formed chiefly by the postorbital process) is preserved. The medial surface of the posterior process (i.e. the region ventral to the postorbital process) is mediolaterally thin and concave where it would have overlapped the lateral surface of the quadratojugal.

**Remarks**

LRF R1556 can be differentiated from the holotype of *L. amicagraphica* (Herne, 2014) in having a more recumbent postorbital process, which also lacks medial and lateral grooves; a grooved contact for the ectopterygoid, and; a straight, grooved maxillary process on the medial surface of the anterior process. It is also potentially differentiated from both *L. amicagraphica* and *Muttaburrasaurus langdoni—*the only other Australian ornithischian with a preserved jugal—in having a truncated and unexpanded posterior process, although, as noted above, this may be the result of breakage in LRF R1556.

Given that the jugal is usually preserved in articulation with the facial skeleton, the medial surface of the jugal has not been described for many neornithischians. However, the articulation between the jugal, maxilla, and ectopterygoid offers some insight into taxonomy of the group (Figs. 7C–7H). In parksosaurids (e.g. *Zephyrosaurus schaffi, O. makelai*) and also *Camptosaurus dispar*, the medial surface of the anterior process bears a medially-projecting and modestly arched maxillary process (Character 39:0; Boyd, 2015). Hadrosauriformes (e.g. *Iguanodon* spp., *Ouranosaurus nigeriensis*) have a similar maxillary process although it is anteromedially projected and more strongly arched (Character 39:2; Boyd, 2015). Dryosaurids (*Dryosaurus altus, Dysalotosaurus lettowvorbecki*) have a rather different arrangement where the maxillary process is straight and grooved (Character 39:1; Boyd, 2015). The shape of the ectopterygoid contact also varies. In Anklyopollexia (*Camptosaurus dispar, Iguanodon* spp.), the contact surface for the ectopterygoid consists of a rounded facet (Character 40:1; Boyd, 2015), whereas in more basal forms (*Dryosaurus altus, Dysalotosaurus lettowvorbecki, O. makelai, Thescelosaurus neglectus, Zephyrosaurus schaffi*), the ectopterygoid inserts into a short, deep groove on the medial surface of the jugal (Character 40:0; Boyd, 2015). Based on these observations, LRF R1556 is most reminiscent of dryosaurids: the maxillary process is straight and grooved with a short, deep groove for the ectopterygoid. However, given the limited taxon sample for these characters, it is unclear whether some of these features are more widespread among non-marginocephalian neornithischians. Other taxonomically-informative features (such as the morphology of the postorbital and lacrimal contacts) are not observable in LRF R1556. We therefore tentatively identify LRF R1556 as a non-ankylopollexian iguanodontian or dryomorphan.

Ankylopollexia Sereno 1986
Ankylopollexia indet.

**Material:** LRF 267, basioccipital-basisphenoid.

**Locality and horizon:** Unknown locality (probably Emu’s field) on the Coocoran opal fields, New South Wales; Wallangulla Sandstone, GCF, Cenomanian, Rolling Downs Group of the Surat Basin.

**Description**

**Figure 8:**
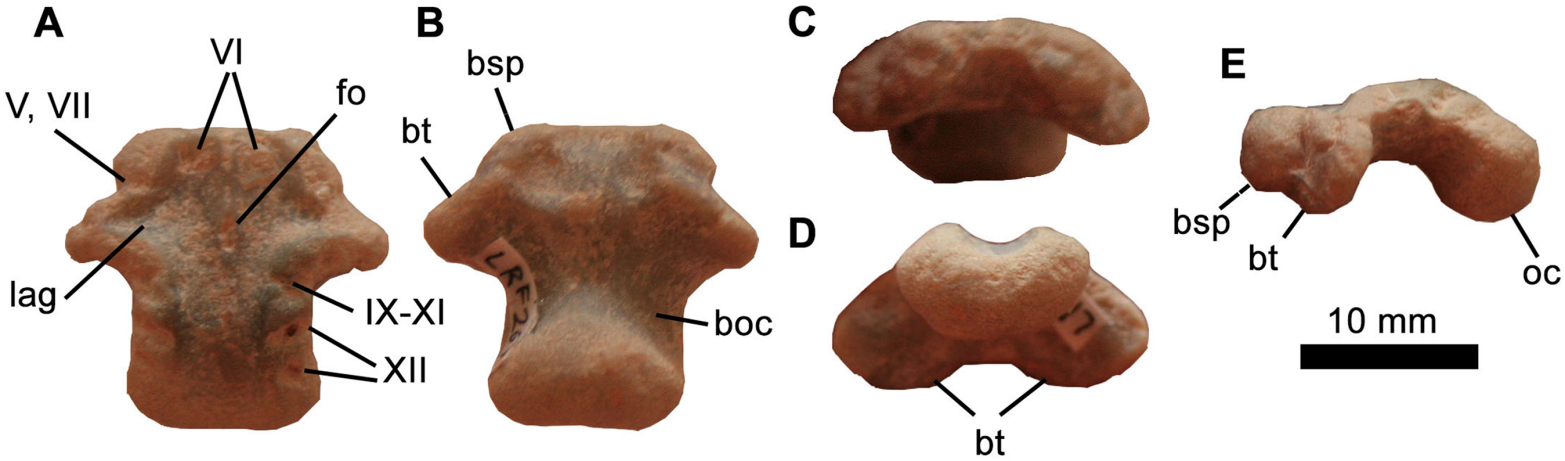
Iguanodontia indet. basioccipital (LRF 267). (A) Dorsal, (B) ventral, (C) anterior, (D) posterior, and (E) left lateral views. Abbreviations: boc, basioccipital; bsp, basisphenoid; bt, basal tubera; for, foramen; lag, lagena; oc, occipital condyle. Photo credit: Phil Bell.

The basioccipital-basisphenoid (LRF 267) consists of a fused basioccipital and basisphenoid; the line of fusion between the two elements has been entirely obliterated (Fig. 8). Overall, the element is slightly mediolaterally broader than it is long. In dorsal view, the anterior margin is flat and straight, as in *Thescelosaurus assiniboiensis* (Brown, Boyd & Russell, 2011), where it would have abutted the basisphenoid. The paired basal tubera are blade-like and in ventral view are oriented posterolaterally. In anterior view they are semicircular, separated by a medial concavity that is as broad as the basal tubera are anteromedially-posterolaterally long. There is no sagittal ridge in this region, which is also the case for *Muttaburrasaurus langdoni* (QM F6140) and *Zalmoxes* spp. (Weishampel et al., 2003; Godefroit, Codrea & Weishampel, 2009) but unlike *Anabisetia saldivai* (MC PVPH-74), *G. cincosaltensis* (MUCPv-208), *Hypsilophodon foxii* (Galton, 1974) and all other non-iguanodontian neornithischians (Boyd, 2014). The posterolateral edges of the basal tubera form the broadest part of the basioccipital. Immediately posterior to the basal tubera, the basioccipital is constricted ventrally and, to a lesser extent, mediolaterally to form a distinct ‘neck’ that leads to the occipital condyle. A distinct neck is absent in *Zalmoxes shqiperorum* and hadrosaurids (Godefroit, Codrea & Weishampel, 2009). The occipital condyle is bulbous and reniform in posterior view. The concave dorsal surface of the occipital condyle forms a significant contribution to the ventral margin of the foramen magnum is it does in most neornithischians. In fact, in dorsal view, the basioccipital contribution to the foramen magnum is approximately 40% of the width of the posterodorsal surface of the basioccipital (Fig. 8D), which is greater than many parksosaurids (e.g. *O. makelai, Oryctodromeus cubicularis, Thescelosaurus neglectus, Zephyrosaurus schaffi* (Boyd, 2014)). This contribution is reduced in more derived iguanodontians (e.g. *Dysalotosaurus lettowvorbecki, R. priscus, Camptosaurus dispar* (Hübner & Rauhut, 2010)) and is lost altogether in *G. cincosaltensis* and *Iguanodon bernissartensis* where the basioccipital is excluded from the foramen magnum by the exoccipitals (Norman, 1980, 1986; Coria & Salgado, 1996). In posterior view, the sutural surfaces for the exoccipitals/opisthotics are dorsomedially inclined. The dorsolateral margins of the basioccipital each bear three notches that would have formed the floors of the canals transmitting the cranial nerves. The anteriormost notch is V-shaped in dorsal view, reflecting the confluence of two minor canals into a common opening for c.n. IX–XI. The two more posteriorly-placed notches are interpreted as the paired openings for c.n. XII. The anterior of these two notches is posteromedially directed (in dorsal view), whereas the posterior one presents as a subcircular ‘dimple’.

The entire dorsal surface of the fused basisphenoid–basioccipital is dominated by a median ‘t’-shaped depression, which forms the floor of the braincase. Within this depression, immediately posterior to the anterior margin of the basisphenoid are two pits, which likely formed the paired openings for cranial nerve VI. At about one-third of the distance from the anterior edge, is a small, elliptical midline fossa (Fig. 8A). The significance of this fossa is unclear, and we are unaware of the presence of this feature in other ornithopods. In line with this fossa in LRF 267 are triangular left and right lateral extensions of the median depression, which formed the floor of the inner ear (in the region of the lagena) leading to the fenestra ovalis. Immediately anterior to this depression, presumably on the lateral edge of the basisphenoid (although the basisphenoid and basioccipital are indistinguishably fused) is a shallow notch interpreted as the floor of the common opening for cranial nerves V and VII.

**Remarks**

Dinosaur basicrania are highly conservative; however, LRF 267 is identifiable as an ornithischian based on its apneumatic, anteroposteriorly elongate form and presumed subhorizontal orientation (based on the orientation of the floor of the endocranial cavity; Fig. 8E). In contrast, the basioccipital in theropods is typically pneumatic and anteroventrally oriented (Paulina Carabajal, 2009, 2011). Many neosauropods possess a basioccipital depression between the basal tubera and the occipital condyle (Wilson, 2002) and most titanosaurs—the most widely documented group of sauropods from the Cretaceous of Australia—also possess a median subcondylar foramen (Díez Díaz, Pereda Superbiola & Sanz, 2011 and references therein), neither of which is present in LRF 267. Eusuchian crocodylomorphs have a strongly ‘verticalised’ basioccipital (Gower & Webber, 1998; Salisbury et al., 2006) and there is no evidence in LRF 267 that the Eustachian system is enclosed by the basisphenoid as it is in crocodilians (and convergently in birds; Gower & Webber, 1998) nor evidence for a foramen between the basioccipital and basisphenoid (median pharyngeal foramen (=median Eustachian foramen)), which is characteristic of Crocodyliformes more broadly (Benton & Clark, 1988).

LRF 267 possesses a mosaic of derived and more basal features: The absence of a ventral keel and a flat floor of the braincase resembles the basioccipital of derived iguanodontians within Ankylopollexia (e.g. *Camptosaurus dispar, I. bernissartensis*). In contrast, a ventral keel is present and the floor of the braincase is also arched in all non-iguanodontian neornithischians (Scheetz, 1999; Boyd, 2014). Dryosaurids (*Dryosaurus altus, Dysalotosaurus lettowvorbecki*) also possess a ventral keel (Janensch, 1955; Galton, 1983; Hübner & Rauhut, 2010). On the other hand, the basioccipital of LRF 267 forms a relatively broad contribution to the foramen magnum, which is the primitive neornithischian condition and unlike what is seen in Iguanodontia. Overall, however, most of the features of LRF 267 allow us to tentatively identify it as an ankylopollexian.

## Phylogenetic Analysis

**Figure 9:**
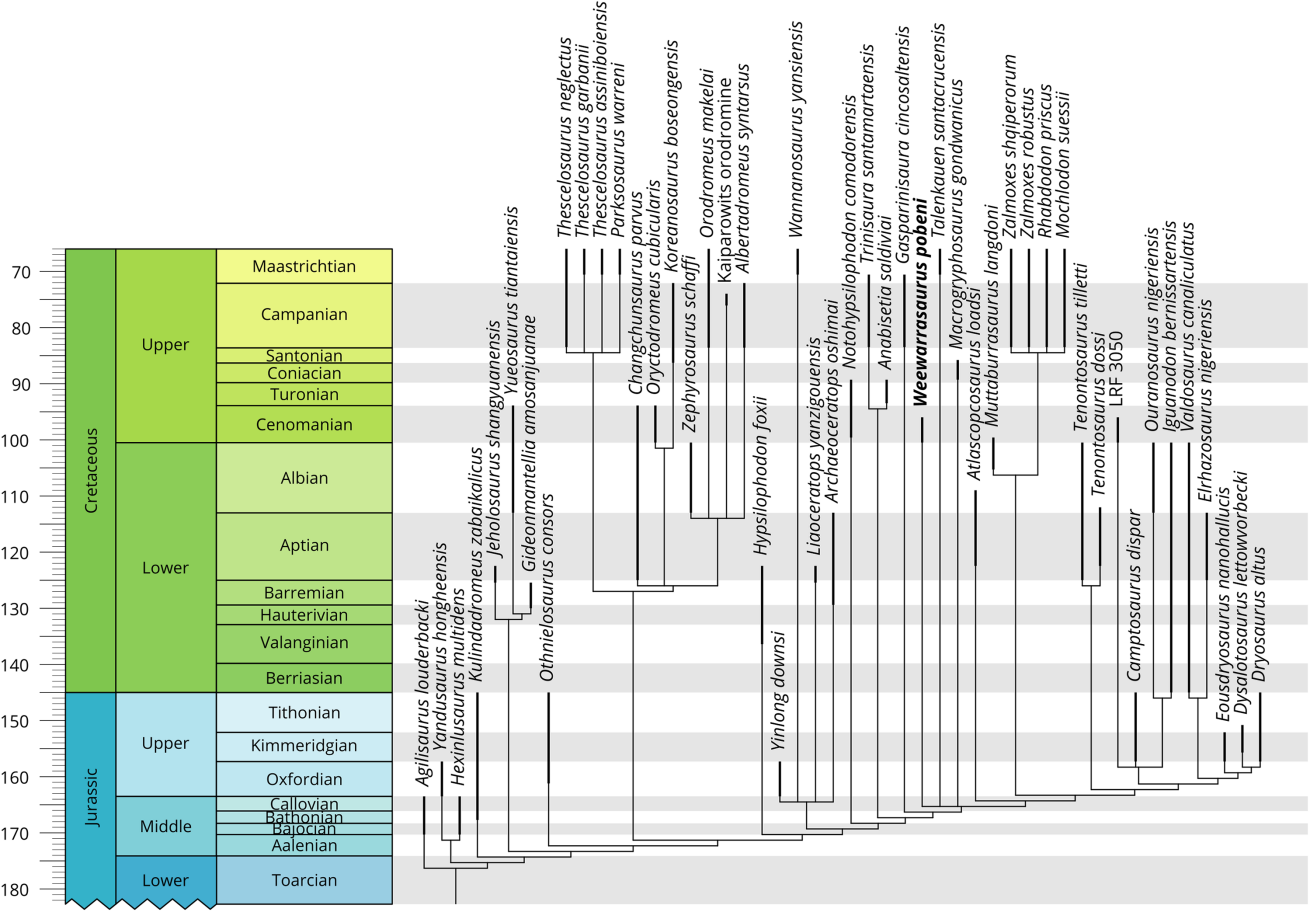
Time calibrated phylogeny for *Weewarrasaurus pobeni* gen. et sp. nov. and Neornithischia. The reduced strict consensus tree was produced from >100,000 MPTs of the modified Boyd (2015) matrix. The black bars indicate maximum stratigraphic ranges for each terminal node. The complete tree is presented in File S2. Geological timescale based on dates provided by the International Commission on Stratigraphy.

The phylogenetic analysis returned more than 100,000 MPTs with a length of 913 steps (CI = 0.342, RI = 0.639). The strict consensus of the MPTs placed *W. pobeni* within a large polytomy of ornithischian taxa (File S2). Within this polytomy, only Thyreophora, (*Jeholosaurus* + *Yueosaurus* + *Gideonmantellia*), Parksosauridae, (*Anabisetia* + *Trinisaura*) and (Dryomorpha + *Tenontosaurus* sp.) resolved as discrete clades. To improve resolution of the strict consensus tree, unstable taxa in the MPTs were identified using iterative PCR (Pol & Escapa, 2009), as implemented in TNT using the pcrprune function, and pruned a posteriori to produce a reduced strict consensus tree. A total of 11 unstable taxa were identified: *Pisanosaurus mertii*, *Lycorhinus angustidens*, *L. amicagraphica*, *Stenopelix valdensis*, *Micropachycephalosaurus hongtuyanensis*, *Q. intrepidus*, *Morrosaurus antarcticus*, *Burianosaurus augustai*, *Mochlodon vorosi*, *Haya griva, Callovosaurus leedsi*. The reduced strict consensus tree resolved *Weewarrasaurus*, together with the elasmarian taxa *Talenkauen* and *Macrogryphosaurus*, in a polytomy with a clade containing *Atlascopcosaurus* and Iguanodontia (i.e. Rhabdodontidae + *Muttaburrasaurus* (i.e. Rhabdodontomorpha, sensu Dieudonné et al., 2016) and *Tenontosaurus* spp. + Dryomorpha (Fig. 9, see File S3 for the complete tree)). Of the four unambiguous synapomorphies diagnosing the aforementioned polytomy (Character 138: 0 → 1, Character 197: 2 → 1, Character 205: 0 → 1, Character 226: 0 → 1), only one is observable in *Weewarrasaurus*: 10 or more ridges present on dentary teeth (Character 138(1)).

## Discussion

A reassessment of neornithischian craniodental remains from the GCF yields new insights into the diversity of ornithopods at Lightning Ridge (Table 1). At least two taxa of small-bodied basal ornithopods (*W. pobeni*, Ornithopoda indet. A), as well as one large-bodied indeterminate Iguanodontia and a small-bodied Ankylopollexia, appear to have coexisted. Earlier work on the GCF identified two morphotypes of ‘hypsilophodontid’ and a third *Muttaburrasaurus*-like taxon (Molnar, 1980a, 1996; Molnar & Galton, 1986). Since the two ‘hypsilophodontids’ observed by Molnar & Galton (1986) were based on femoral morphology, they cannot be matched to the taxa recognised here based on craniodental remains. Nevertheless, with the exception of the possible ankylopollexian identified here, our results corroborate those of Molnar (1980a, 1996) and Molnar & Galton (1986).

Without more complete material, it is impossible to determine whether the larger isolated teeth (AM F81865, AM F119236, LRF 492, LRF 660, QM F14421) belong to one or as many as four separate taxa; however, all appear to be assignable to a relatively large-bodied (at least in comparison to the craniodental remains described here) non-dryomorphan iguanodontian. The isolated jugal (LRF R1556) displays some notable dryosaurid characteristics in the forms of the ectopterygoid contact and the maxillary process, but also potentially shares with the basal iguanodontian *G. cincosaltensis* a truncated posterior process. Given the uncertain distribution of characters related to the ectopterygoid contact and maxillary process (the medial surface of neornithischian jugals are rarely described/visible), a dryosaurid affinity for LRF R1556 cannot be confirmed and a non-ankylopollexian iguanodontid identity is probably more tenable. Regardless, the jugal cannot be unequivocally assigned to any of the dental morphotypes described here in the absence of overlapping material.

The most derived ornithopod remains that can be identified from the isolated material pertain to the isolated basicranium (LRF 267). Based on the straight (not arched) floor of the braincase and the clear absence of a ventral keel, we assign this specimen to Ankylopollexia. The substantial contribution of the basioccipital to the foramen magnum is a potentially more primitive character, however, and we acknowledge that this assignment is liable to change with more complete material. Despite its small size, the identification of LRF 267 as an Ankylopollexia is at least in keeping with Agnolin et al.’s (2010) identification of *Muttaburrasaurus langdoni* as a member of the Styracosterna, the only Australian taxon assigned to that clade. Agnolin et al.’s (2010) assignment—based on a significant number of anatomical features—, however, has not been followed in more recent phylogenetic analyses, where *Muttaburrasaurus* has been recovered as non-dryomorphan Iguanodontia (McDonald, 2012; Boyd, 2015; Dieudonné et al., 2016; Madzia, Boyd & Mazuch, 2018). Given that most studies that have included *Muttaburrasaurus* in a phylogenetic analysis have done so based on casts of the poorly-preserved holotype, pending the full redescription of *Muttaburrasaurus* (M. Herne et al., 2018, unpublished data) we regard the position of *Muttaburrasaurus* as equivocal at this stage.

The recent identification of ankylosaur material from the GCF (Bell, Burns & Smith, 2017) also warrants some consideration in the identification of the isolated teeth described here; however, these can be readily differentiated from the teeth of ankylosaurians. Ankylosaurid cheek teeth (maxillary and dentary) differ from neornithischians in having primary and secondary ridges that do not extend to the cingulum (Kirkland, 1998; Galton, 2007). The cingulum is also more pronounced in ankylosaurids, which gives rise to a stronger distinction between the cingulum and the laterally compressed crown (Coombs & Maryańska, 1992). As in other thyreophorans (Norman, Witmer & Weishampel, 2004b), the root is straight and unlike the curved roots of many ornithopods including several of those described here.

In contrast to the relatively rich ornithopod fauna from the GCF, no more than two taxa have been recorded from any one of the roughly coeval formations in Queensland to the north. The holotype of *Muttaburrasaurus langdoni* comes from the Mackunda Formation (late Albian–early Cenomanian) whereas *Muttaburrasaurus* sp. (QM F14921) comes from the underlying Allaru Mudstone and has been regarded as a distinct species by some authors (Molnar, 1996, Agnolin et al., 2010). Only a single small-bodied ornithopod (an isolated tooth of a non-iguanodontian ornithopod, based on a prominent and centrally-placed primary ridge, and well-developed secondary ridges that terminate in a single denticle) has been described from Queensland from the upper part (Cenomanian) of the Winton Formation (Hocknull & Cook, 2008). Ichnological evidence from the Winton Formation at Lark Quarry (which straddles the Cenomanian–Turonian boundary; Tucker et al., 2013) corroborates the presence of a single small-bodied neornithischian trackmaker (*Wintonopus latomorum*) and adds to the faunal list a large-bodied ornithopod (*Amblydactylus* cf. *A. gethingi*; Thulborn & Wade, 1984; Romilio & Salisbury, 2011; Romilio, Tucker & Salisbury, 2013). In contrast to the Queensland fauna, at least four (but as many as five according to Herne (2014)) taxa are known from the Eumeralla and temporally-equivalent Wonthaggi formations in Victoria, including a basal neornithischian (*L. amicagraphica*) and small-bodied basal ornithopods (*Atlascopcosaurus loadsi, Diluvicursor pickeringi, Q. intrepidus*). Large-bodied iguanodontians are notably absent.

The reason(s) behind such faunal differences between these three regions is speculative although it was probably influenced—at least in part—by palaeoenvironmental gradients between the north and south of the country. Sauropods, in particular, appear to have been strongly influenced by climate, being found in palaeolatitudes no higher than 66° (north or south) and in greater diversity at lower latitudes (Poropat et al., 2016 and references therein). In the Winton Formation, sauropods are the most commonly recovered dinosaur remains (Hocknull et al., 2009; Poropat et al., 2015, 2016), whereas they are unknown from the well-sampled deposits in Victoria. On the other hand, sauropods from the GCF—represented by rare isolated teeth at Lightning Ridge—appear to have been at about their southern limit (Molnar & Salisbury, 2005; Mannion et al., 2012; Poropat et al., 2016). The GCF, which during this time resided at ∼60°S, was subjected to a highly seasonal climate with extended dark periods during the winter months, although a diverse mesoreptile fauna (crocodyliforms and turtles; Molnar, 1980b; Molnar & Willis, 2001; Smith, 2010; Smith & Kear, 2013) suggests that average minimum temperatures may not have dipped much below ∼5 °C (Markwick, 1998). Whereas the Griman Creek and Winton formations share a titanosauriform fauna to the exclusion of the Eumeralla and Wonthaggi formations, the Griman Creek and Eumeralla/Wonthaggi formations appear to share a higher diversity of small-bodied neornithischians. On the other hand, large-bodied *Muttaburrasaurus-*like iguanodontians are absent from the Victorian localities and appear restricted to the Griman Creek and Winton formations. Of course, whether or not titanosauriform sauropods and large-bodied iguanodontians ranged southwards to Victoria after the Albian (i.e. following deposition of the Eumeralla/Wonthaggi formations) is unknown due the lack of appropriately-aged strata. Similarly, taphonomic considerations and relatively poor sampling cannot be ruled out for the apparent dearth of small ornithopods in the Winton Formation (and related formations in Queensland; e.g. Allaru Mudstone), which commonly preserves large-bodied vertebrates (e.g. sauropods, megaraptoran theropods). Other terrestrial vertebrate components (e.g. megaraptoran theropods, ankylosaurians) are ubiquitous in eastern Australia and may not have been as strongly influenced by climatic gradients, although it is notable that crocodyliforms were also present across eastern Australia at the time (Molnar, 1980; Salisbury et al., 2006).

## Conclusions

The GCF at Lightning Ridge supported a diverse ornithopod fauna consisting of two-to-three small-bodied non-iguanodontian ornithopods (including *Weewarrasaurus pobeni* gen. et sp. nov.), at least one indeterminate iguanodontian, and a possible ankylopollexian. The new taxon, *W. pobeni* gen. et sp. nov., is recovered within a polytomy of Gondwanan taxa typically considered elasmarians, including *Anabisetia, Gasparinisaura, Macrogryphosaurus,* and *Trinisaura*. The position of *Weewarrasaurus* within this polytomy, however, hinges on a single character, therefore it’s position is likely to change with the future discovery of additional phylogenetically-informative material. These results support those of previous studies that favour a general abundance of small-bodied basal ornithopods in Early to mid-Cretaceous high-latitude localities of southeastern Australia, including the Wonthaggi and Eumeralla formations in Victoria and the GCF in New South Wales. Although future discoveries have the potential to alter these interpretations, these considerations suggest that the GCF at Lightning Ridge occupied a ‘meeting point’ between more northern sauropod-dominated faunas and the ornithopod-dominated faunas in the south.

## Supplemental Information

10.7717/peerj.6008/supp-1 Supplemental Information 1 Complete modified matrix of Madzia et al. (2017).

10.7717/peerj.6008/supp-2 Supplemental Information 2 Strict consensus of the >100,000 MPTs from the modified Boyd (2015) matrix including Weewarrasaurus.

10.7717/peerj.6008/supp-3 Supplemental Information 3 Reduced strict consensus of the >100,000 MPTs from the modified Boyd (2015) matrix including Weewarrasaurus, following a posterior pruning of 15 unstable taxa.

## Institutional Abbreviations

AM: Australian Museum, Sydney, New South Wales, Australia
LRF: Australian Opal Centre, Lightning Ridge, New South Wales, Australia
MCF-PVPH: Museo Municipal ‘Carmen Funes’, Plaza Huincul, Neuquén, Argentina
NMV: Museum Victoria, Melbourne, Victoria, Australia
QM: Queensland Museum, Hendra, Queensland, Australia.

## Additional Information and Declarations

### Data Availability

The following information was supplied regarding data availability: Bell, Phil; Brougham, Tom; Smith, Elizabeth; Herne, Matthew (2018): CT data accompanying the paper ‘Ornithopod diversity in the Griman Creek Formation (Cenomanian), New South Wales, Australia.’ Part 1 of 3. figshare. Fileset. https://doi.org/10.6084/m9.figshare.6959486.v1 Bell, Phil; Herne, Matthew; Brougham, Tom; Smith, Elizabeth (2018): CT data accompanying the paper ‘Ornithopod diversity in the Griman Creek Formation (Cenomanian), New South Wales, Australia.’ Part 2 of 3. figshare. Fileset. https://doi.org/10.6084/m9.figshare.6962816.v1 Bell, Phil; Herne, Matthew; Brougham, Tom; Smith, Elizabeth (2018): CT data accompanying the paper ‘Ornithopod diversity in the Griman Creek Formation (Cenomanian), New South Wales, Australia.’ Part 3 of 3. figshare. Fileset. https://doi.org/10.6084/m9.figshare.6959372.v1 All described specimens are accessioned in recognised museum collections: Australian Museum, Sydney, New South Wales, Australia (AM), Australian Opal Centre, Lightning Ridge, New South Wales, Australia (LRF), Queensland Museum, Hendra, Queensland, Australia (QM). LRF 267, LRF R1556, LRF 492, AM F119236 (QMF 14420, a cast), AM F81865, AM F119282 (QMF 14421, a cast), AM F112862, LRF 660, AM F105667, AM F106297, LRF 766, LRF 3067 (holotype of *Weewarrasaurus pobeni* gen. et sp. nov.).

### New Species Registration

The following information was supplied regarding the registration of a newly described species: Publication LSID: urn:lsid:zoobank.org:pub:E8AFFE1D-FACA-4A69-BAA9-5FF6EE1F2EAD Genus name Weewarrasaurus gen. nov. LSID: urn:lsid:zoobank.org:act:BD3BF258-DBC2-4D37-8143-07C24BA8850C Species name *Weewarrasaurus pobeni* sp. nov. LSID: urn:lsid:zoobank.org:act:4CA9B960-AD74-4748-B22D-DDEF6BF2498C.
