# Supplementary figures and images for: Ornithopod diversity in the Griman Creek Formation (Cenomanian), New South Wales, Australia
Source: PeerJ. 2018 Dec 4;6:e6008. doi: 10.7717/peerj.6008 (PMC6284429; doi:10.7717/peerj.6008)

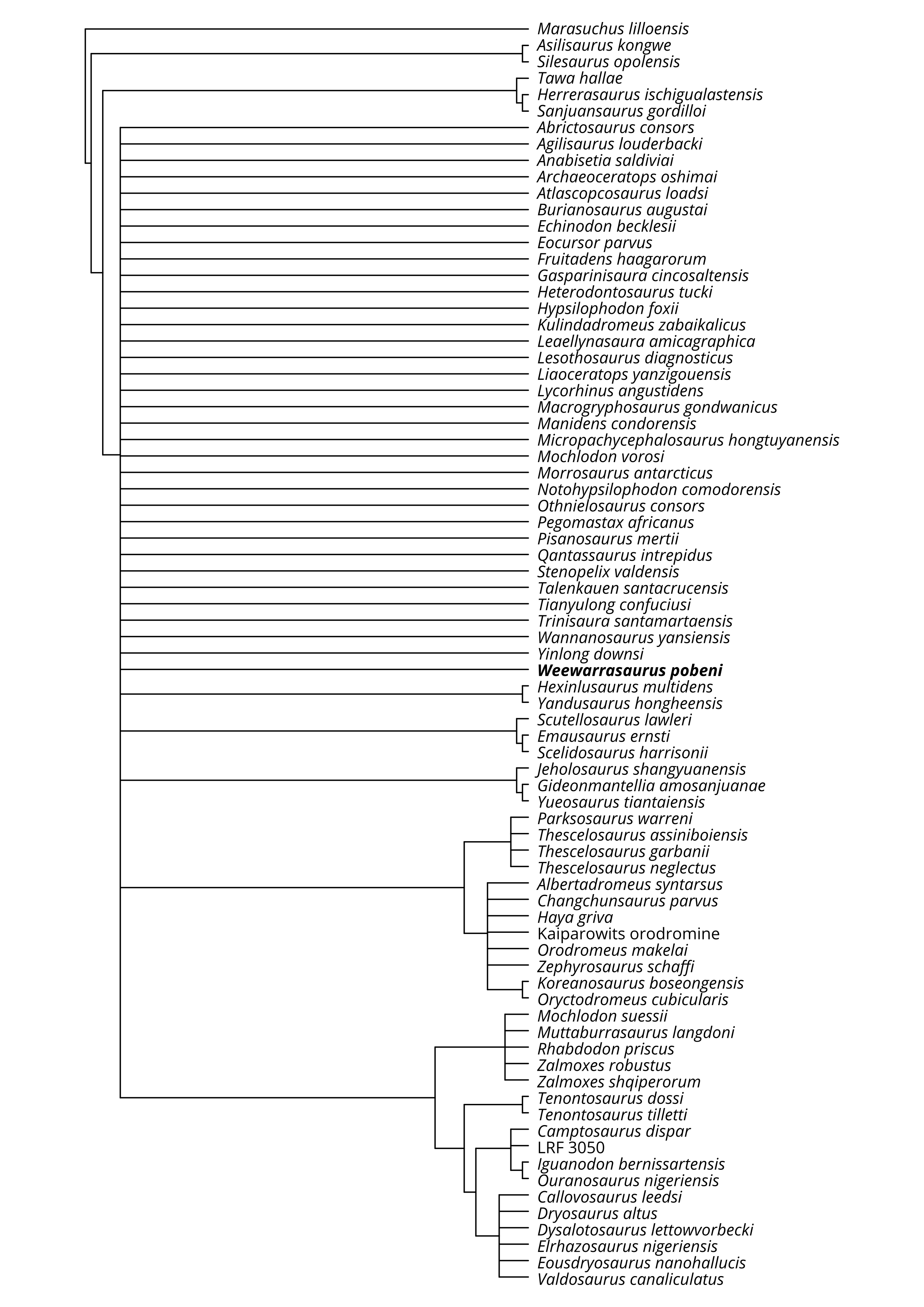

Supplement: Supplemental Information 2 [file peerj-06-6008-s002.png]

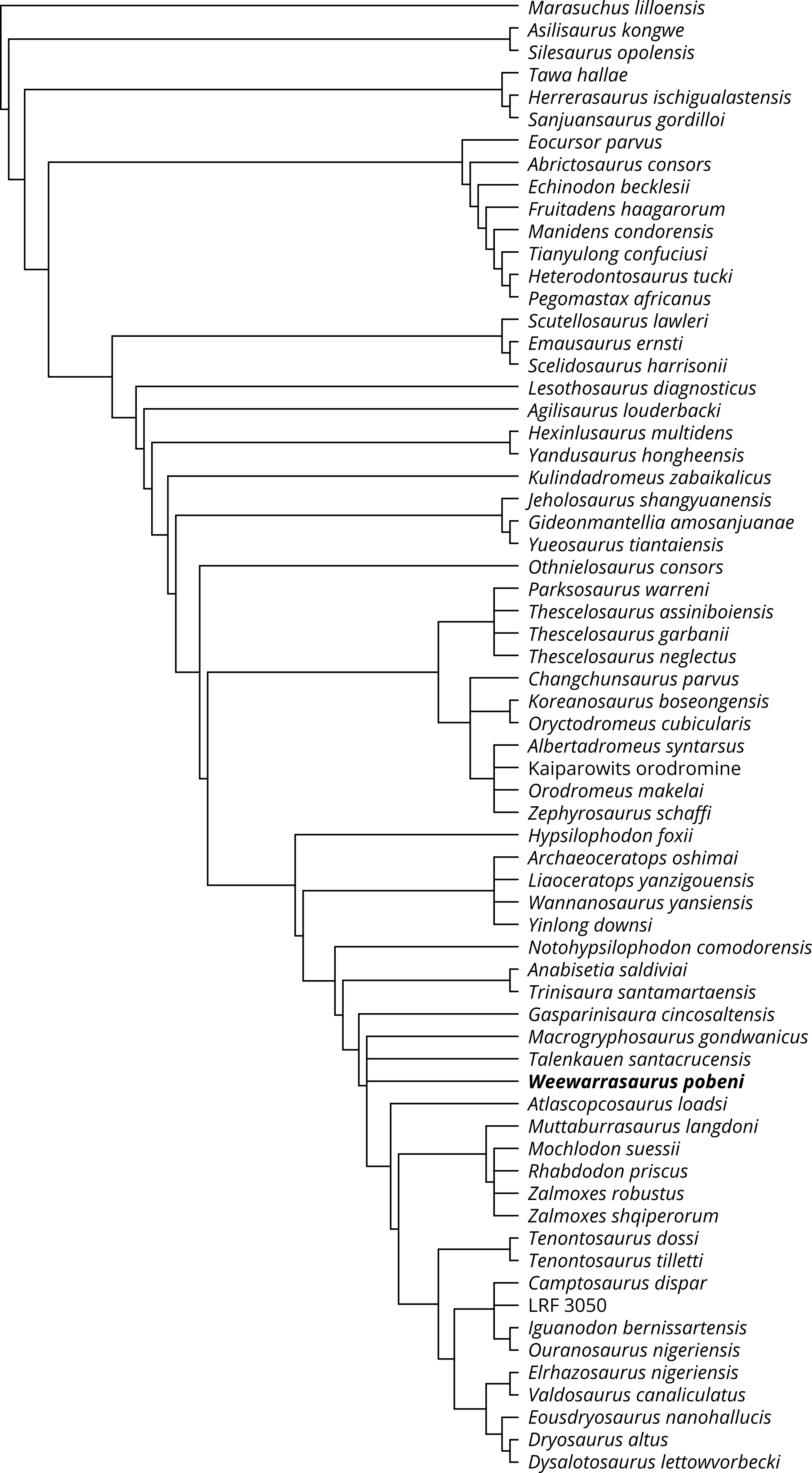

Supplement: Supplemental Information 3 [file peerj-06-6008-s003.png]
